# Li_4.3_AlS_3.3_Cl_0.7_: A Sulfide–Chloride Lithium Ion Conductor with Highly Disordered Structure and Increased Conductivity

**DOI:** 10.1021/acs.chemmater.1c02751

**Published:** 2021-11-10

**Authors:** Jacinthe Gamon, Matthew S. Dyer, Benjamin B. Duff, Andrij Vasylenko, Luke M. Daniels, Marco Zanella, Michael W. Gaultois, Frédéric Blanc, John B. Claridge, Matthew J. Rosseinsky

**Affiliations:** † Department of Chemistry, University of Liverpool, Crown Street, L69 7ZD Liverpool, United Kingdom; ‡ Stephenson Institute for Renewable Energy, University of Liverpool, Peach Street, L69 7ZF Liverpool, United Kingdom; § Leverhulme Research Centre for Functional Materials Design, Materials Innovation Factory, University of Liverpool, L69 7ZD Liverpool, United Kingdom

## Abstract

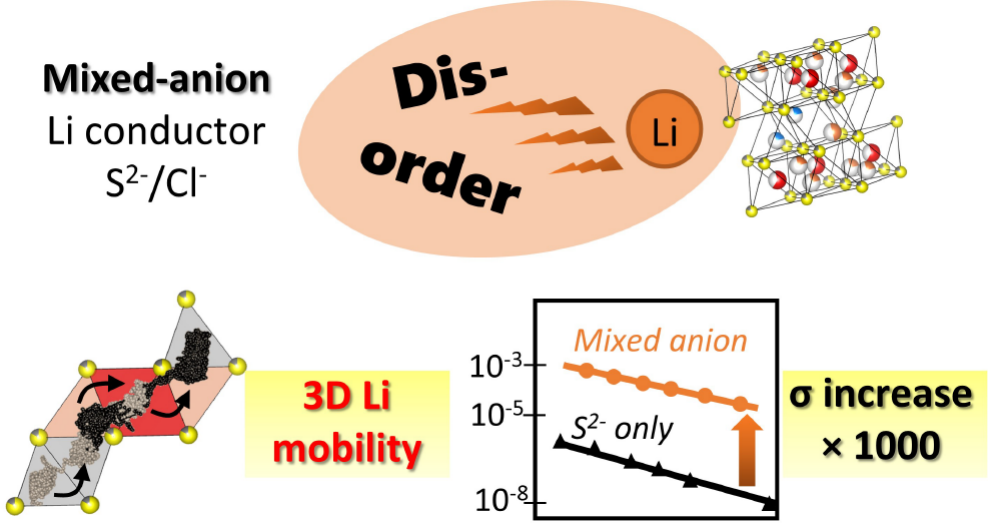

Mixed anion materials and anion doping are very promising strategies to improve solid-state electrolyte properties by enabling an optimized balance between good electrochemical stability and high ionic conductivity. In this work, we present the discovery of a novel lithium aluminum sulfide–chloride phase, obtained by substitution of chloride for sulfur in Li_3_AlS_3_ and Li_5_AlS_4_ materials. The structure is strongly affected by the presence of chloride anions on the sulfur site, as the substitution was shown to be directly responsible for the stabilization of a higher symmetry phase presenting a large degree of cationic site disorder, as well as disordered octahedral lithium vacancies. The effect of disorder on the lithium conductivity properties was assessed by a combined experimental–theoretical approach. In particular, the conductivity is increased by a factor 10^3^ compared to the pure sulfide phase. Although it remains moderate (10^–6^ S·cm^–1^), ab initio molecular dynamics and maximum entropy (applied to neutron diffraction data) methods show that disorder leads to a 3D diffusion pathway, where Li atoms move thanks to a concerted mechanism. An understanding of the structure–property relationships is developed to determine the limiting factor governing lithium ion conductivity. This analysis, added to the strong step forward obtained in the determination of the dimensionality of diffusion, paves the way for accessing even higher conductivity in materials comprising an *hcp* anion arrangement.

## 1 Introduction

In the past decades, solid-state electrolytes have grown as a promising solution for preventing safety hazards originating from liquid electrolyte solvent flammability in lithium batteries. Overcoming the intrinsic lower ionic conductivity of solids compared to liquids as well as meeting the requirement for electrochemical stability *vs.* electrodes are the two main challenges for finding a viable candidate. Incredible progress in this direction has been made in recent years.^1−3^ The room temperature lithium conductivity target of 10^–3^ S·cm^–1^ has now been met in different families of materials, including garnet type Li_6.55+*y*_Ga_0.15_La_3_Zr_2–*y*_Sc_*y*_O_12_ (1.8 × 10^–3^ S·cm^–1^),^4^ glass–ceramic 70 Li_2_S–30 P_2_S_5_ (mol %) (1.7 × 10^–2^ S·cm^–1^),^5^ thio-LISICON Li_9.54_Si_1.74_P_1.44_S_11.7_Cl_0.3_ (2.5 × 10^–2^ S·cm^–1^),^6^ and Li_3_YBr_6_ (1.7 × 10^–3^ S·cm^–1^).^7^ However, these materials still suffer from limitations such as high production costs (oxides garnets), sensitivity to moisture and air (sulfides), poor compatibility to cathode materials (hydrides), and low oxidation potential (halides). Developing innovative exploratory chemistry to access new functional lithium solid-state electrolytes is therefore still very much at stake.

Research on mixed anion materials is expanding significantly and presents an original way to modulate structure and properties in many fields of material science.^8,9^ As for cation substitution, which has been extensively studied, anion doping strategies have been shown to be very promising for the stabilization of disordered phases and improved conductivity properties.^10−15^ Examples comprise the lithium argyrodite family, for which the pure sulfide phase Li_7_PS_6_ presents an orthorhombic unit cell at room temperature, and the incorporation of the halide anion leads to the stabilization of the cubic polymorph Li_6_PS_5_*X* (*X* = Cl, Br, I) with high lithium mobility.^16^ High alkali conductivity was also achieved in the Li*X*–LiBH_4_ system (*X* = Br, I),^15^ through the stabilization, at room temperature, of the hexagonal polymorph with defect wurtzite structure showing a conductivity increase of 2 orders of magnitude.^17^

Mixed anion chemistry can typically be used to optimize properties by combining advantages of more than one family of materials. While oxides often show better atmospheric and electrochemical stability, sulfide electrolytes present among the highest reported lithium conductivities, thanks to their high polarizability and increased cation–anion bond covalency compared to more electronegative anions. For instance, oxysulfide glass of composition Li_2_S–SiS_2_–Li_3_PO_4_ has been reported as an attractive solution, with improved electrochemical stability compared to the pure sulfide counterparts, while maintaining good lithium conductivity.^18^ Halides present the advantages of being highly stable against Li metal, being less prone to oxidation compared to sulfides,^2,7^ and have recently been identified, through a data driven approach, as very likely to yield high performance outlier discovery.^19^ Moreover, as halide anions have a lower charge than sulfide anions, the halide for sulfide substitution will enable cation off-stoichiometry, favorable for conductivity. Cl^–^ in particular, which has an ionic radius close to that of S^2–^,^20^ favors mixed occupancy on the anionic sites and, hence, disorder. In addition to the lithium argyrodite family cited above, two phases in the Li_2_S–Li_2_PS_5_–LiI phase field, Li_4_PS_4_I^21^ and Li_7_P_2_S_8_I,^22^ were reported with enhanced lithium conductivity and good electrochemical stability (10 V *vs*. Li/Li^+^ for Li_7_P_2_S_8_I).^22,23^ Other efforts focus on the preparation of sulfide–halide glass^24,25^ or on the introduction of a small amount of chlorine into sulfide materials, such as Li_9.54_Si_1.74_P_1.44_S_11.7_Cl_0.3_, which is among the best lithium solid electrolytes.^6^

The exploration of sulfide–chloride materials for solid electrolyte application is therefore a promising yet still little-explored area. In this work, we report on the synthesis of a new lithium and aluminum mixed anion sulfide–chloride material of composition Li_5–*y*_Al_1+(*y*–*x*)/3_S_4–*x*_Cl_*x*_ (*x* = 0.5–0.7; *y* = 0.5–1) with a highly disordered structure. The in-depth characterization of Li_4.3_AlS_3.3_Cl_0.7_ shows considerable improvement of the lithium conductivity properties compared to the monoanionic sulfide parent phase. The effect of mixed anion and disorder on the ionic conductivity is studied by a combined experimental–computational approach.

## 2 Experimental Section

### 2.1 Synthesis

Samples with compositions Li_5–*x*_AlS_4–*x*_Cl_*x*_ (*x* = 0.3; 0.5; 0.7; 1), Li_5_Al_1–*x*/3_S_4–*x*_Cl_*x*_ (*x* = 0.15; 0.5; 0.7), Li_3–*x*_AlS_3–*x*_Cl_*x*_ (*x* = 0.05; 0.1; 0.2; 0.4) and Li_3_Al_1–*x*/3_S_3–*x*_Cl_*x*_ (*x* = 0.2; 0.4; 0.5) were made by solid state synthesis. Stoichiometric amounts of Li_2_S (Merck, 99.98%), Al_2_S_3_ (Alfa Aesar, 99+%), and LiCl (Merck, 99.99%) powder were weighted in order to yield a total mass of powder of 300 mg. For the title compound Li_4.3_AlS_3.3_Cl_0.7_, 158 mg of Li_2_S, 108 mg of Al_2_S_3_, and 34 mg of LiCl were weighted. Powders were combined and mixed thoroughly in an agate mortar for 15 min, transferred in an alumina crucible, and then sealed in a quartz tube with Ar under a pressure of 10^–4^ mbar. The tube containing the sample was heated to 700 °C at a ramp rate of 5 °C·min^–1^, held at 700 °C for 12 h, and then cooled to room temperature at a ramp rate of 5 °C·min^–1^. The resulting powder was then manually ground in order to obtain a fine powder. Precursors and resulting powders were handled in an Ar-filled glovebox.

### 2.2 Elemental Analysis

Elemental analysis of Li_4.3_AlS_3.3_Cl_0.7_ was performed by Mikroanalytishes Labor Pascher at Remagen-Bandorf, Germany, following the procedure described below:

#### Chloride

About 3 mg (precisely weighed) was dissolved in diluted aqueous H_2_O_2_ solution. After filling up to a precise volume, chloride was detected by ion chromatography using a Compact IC 761- (Metrohm) instrument. (The result was also checked by combustion/ion chromatography).

##### Aluminum, Lithium, and Sulfur

The dissolution of the sample was performed with HNO_3_/HCl under pressure at 180 °C. The elements were detected by ICP-AES (inductively coupled plasma atomic emission spectrometry; iCap 6500, Thermo Fisher Scientific).

### 2.3 Diffraction

Routine analysis of phase purity and lattice parameters was performed on a Bruker D8 Advance diffractometer with a monochromated Cu source (Kα1, λ = 1.54060 Å) or on a Rigaku SmartLab diffractometer with monochromated Mo source (Kα1, λ = 0.70932 Å) in powder transmission Debye–Scherrer geometry (capillary) with sample rotation. Synchrotron X-ray diffraction (SXRD) was performed at the I11 beamline at Diamond Light Source (Oxfordshire, U.K.), with an incident wavelength of 0.825186 Å using a wide-angle position sensitive detector and samples sealed in Ø = 0.5 mm glass capillaries to prevent air exposure. Time-of-flight (ToF) neutron powder diffraction (NPD) data was collected at room temperature using the Polaris instrument at the ISIS neutron source (Oxfordshire, U.K.). The sample was loaded in a Ø = 6 mm vanadium cylindrical can and sealed in an argon-filled glovebox.

The structural models were refined by the Rietveld method as implemented in the FullProf suite.^26^ Peak shapes were modeled using the Thompson–Cox–Hastings function and the T.O.F. pseudo-Voigt back-to-back exponential function with spherical harmonic expansion for SXRD and NPD data, respectively. All uncertainties were increased by Berar’s factor^27^ (3.2, 5.9, 4.7, 4.6, and 3.1 for SXRD, NPD Bank 2, NPD Bank 3, NPD Bank 4, and NPD Bank 5, respectively, according to FullProf).

### 2.4 AC Impedance Spectroscopy

Pellets were made by uniaxially pressing 30–100 mg of powder in a 5 or 8 mm diameter cylindrical steel die at a pressure of 125 MPa. Pellets were then sintered at 700 °C for 12 h in an evacuated quartz tube. A relative density of 85% was obtained by this method. After the sintering treatment, the pellets showed a black surface, which is attributed to a surface reduction reaction due to the reducing sintering conditions. Prior to electrode deposition, the pellets were polished with sand paper to retrieve a cleaner surface.

AC impedance measurements were performed using an impedance analyzer (Solartron 1296 dielectric interface coupled with the Solartron 1255B frequency response analyzer) in the frequency range from 1 MHz to 100 mHz (with an amplitude of 50 mV). Silver paint (RS silver conducting paint 186-3600), brushed on both sides of the pellet and dried under vacuum at room temperature, was used as ion blocking electrodes. Variable temperature conductivity measurements were carried out under argon (flow rate 50 mL·min^–1^), using a custom built sample holder, in the temperature range 25–125 °C. The impedance spectra were fitted with an equivalent circuit using the ZView2 program.^28^

### 2.5 Raman Spectroscopy

Raman spectra were collected using an inVia Reflex Qontor Confocal Raman Microscope from Renishaw with a laser wavelength of 633 nm. Air sensitive powder samples were sealed in borosilicate glass capillaries inside an argon filled glovebox with level of oxygen and residual moisture smaller than 0.1 ppm. Spectra background removal, data analysis, and plotting was performed using Origin.

### 2.6 NMR

Li_4.3_AlS_3.3_Cl_0.7_ was packed into a 3.2 mm zirconia rotor in an Ar-filled glovebox and ^6^Li and ^27^Al magic angle spinning (MAS) NMR spectra recorded using a 3.2 mm HXY MAS probe in double resonance mode on a 20 T Bruker NEO solid-state NMR spectrometer under MAS at a rate of ω_r_/2π = 20 kHz. ^6^Li spectra were recorded with a pulse length of 3 μs at a radiofrequency (rf) field amplitude of ω_1_/2π = 83 kHz. ^27^Al spectra were obtained with a short pulse angle of 30° of duration 0.2 μs duration at an rf amplitude of ω_1_/2π = 50 kHz. The ^27^Al triple quantum magic-angle spinning (MQMAS)^29^ was obtained with a *z*-filtered sequence^30^ and using rf field amplitudes of ω_1_/2π = 50 kHz for the excitation and reconversion pulses and 20 kHz for the selective 90° pulse. All data acquisitions were quantitative using recycle delays longer than five times the spin–lattice relaxation times, *T*_1_ (measured using a standard saturation recovery sequence). All ^6^Li and ^27^Al shifts were referenced to 10 M LiCl in D_2_O and 0.1 M Al(NO_3_)_3_ in H_2_O at 0 ppm, respectively.

### 2.7 Thermodynamic Phase Stability Calculations

Starting from the experimentally established ordered crystal structure of Li_3_AlS_3_,^31^ we applied the crystal structure prediction code ChemDASH to establish a model of an ordered Cl-doped crystal structure Li_13_Al_3_S_10_Cl_2_ (corresponding to Li_4.3_AlS_3.3_Cl_0.7_). Geometry optimization of the structures was performed with density functional theory (DFT) as implemented in VASP:^32^ with 700 eV for kinetic energy cutoff of the plane waves, PBE pseudopotentials,^33^ and 5 × 5 × 5 k-points grid until forces on atoms were less than 0.001 eV/Å.

From the disordered structures Li_4.3_AlS_3.3_Cl_0.7_ obtained experimentally, we created two disordered structural analogues with compositions Li_5_AlS_4_ and Li_3_AlS_3_: (i) the mixed S/Cl site was replaced by fully occupied S sites only, (ii) in Li_5_AlS_4_, the octahedral lithium site, Li2, was set to a full occupancy, and (iii) in Li_3_AlS_3_ (= Li_4_Al_4/3_S_4_), the site occupancy factors (*sof*) of Al and Li1 were set to 0.33 and 0.42, respectively. This enabled us to match the stoichiometries of both compounds. We then created supercells of the three disordered compounds^34^ and ranked all possible atomic configurations according to their Coulomb energy. For the top 100 structures in this list, we performed DFT geometry optimization and identified the lowest energy structures.

The Gibbs’ free energy of the ordered and disordered structures of Li_4.3_AlS_3.3_Cl_0.7_, Li_5_AlS_4_, and Li_3_AlS_3_ was calculated as
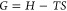
1where *H* is enthalpy of a structure calculated with DFT, *T* is temperature, and *S* is entropy of mixing (configurational entropy) calculated as follows:
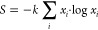
2where *x*_*i*_ is the mole concentration of the *i*th component (atomic species) in the structure and *k* is the Boltzmann constant.

### 2.8 Ab Initio Molecular Dynamics (AIMD)

Two structures were generated as starting points for AIMD calculations. The first structure was chosen from 537 508 symmetrically inequivalent orderings of cations and anions generated using SimDope.^35^ Configurations with composition Li_13_Al_3_S_10_Cl_2_ (Li_4.33_AlS_3.33_Cl_0.67_) were generated from a 2 × 2 × 2 supercell of the experimental structure and avoiding having short Li–Li distances. From these, the 1797 structures in space group *Cm* were chosen to optimize with DFT since they were the highest symmetry structures. Geometry optimization was performed using VASP^36^ with the PBE functional,^33^ a plane wave cutoff energy of 600 eV and a 2 × 2 × 1 *k*-point grid until all forces fell below 0.02 eV Å^–1^. The lowest energy structure was then taken and used to generate a larger supercell with total composition Li_104_Al_24_S_80_Cl_16_ which was used to initialize the AIMD calculations.

The second structure was produced in a similar way by generating 45 513 structures using SimDope but starting with the metal and vacancy ordering present in the structure of Li_4.4_Al_0.4_Ge_0.6_S_4_.^37^ The 728 structures with higher symmetry than *P*1 were optimized as above, and the lowest energy structure was once again chosen to generate a larger supercell with total composition Li_104_Al_24_S_80_Cl_16_ for AIMD calculations.

Fixed cell AIMD calculations were carried out with the PBE functional,^33^ a plane wave cutoff energy of 600 eV and Γ-point only sampling of reciprocal space. A time step of 0.5 fs was used throughout. An initial temperature ramp from 0–400 K was carried out for 4 ps, followed by an equilibration period of 10 ps at 400 K in which temperature was controlled by velocity scaling at every step. A production run of 100 ps was then carried out for both structures at 400 K using a Nosé thermostat.^38^

### 2.9 Maximum Entropy Method (MEM)

The maximum entropy method applied to diffraction data consists of optimizing the reconstruction of the scattering density from the observed structure factors by finding the maximum of the informational entropy under several constraints through an iterative procedure.^39^ MEM is a powerful tool for reconstructing scattering density from incomplete and/or noisy data systems and limits termination effects obtained through usual Fourier synthesis, particularly important in disordered systems.^40^ MEM applied to neutron diffraction data is useful to shed light on the position of light elements, such as Li, poorly visible with X-rays, but presenting a large enough neutron scattering length. This method was recently used to describe conduction pathways in several ionic conductors of lithium^41,42^ and oxygen^43^ in particular. The maximum entropy calculation was performed with the program Dysnomia^44^ using an input file containing observed structure factors from the NPD data of Bank 4 and generated by FullProf.^26^ Visualization of nuclear densities and extraction of 2D displays was then performed in the program Vesta.^45^ Because the ^7^Li scattering length is negative (*b*_Li_ = −2.22 fm), visualization of negative levels is performed to view Li positions within the structure.

## 3 Results and Discussion

### 3.1 Synthesis and Structure Determination

We studied chloride for sulfur substitution in recently reported compounds within the Li–Al–S phase field: Li_5_AlS_4_^46^ and Li_3_AlS_3_.^31^ The structure of these materials can be described as an *hcp*-type packing of sulfur anions within which cations occupy tetrahedral (Li and Al) and octahedral sites (Li only) in a highly ordered pattern.^31,46^

**Figure 1:**
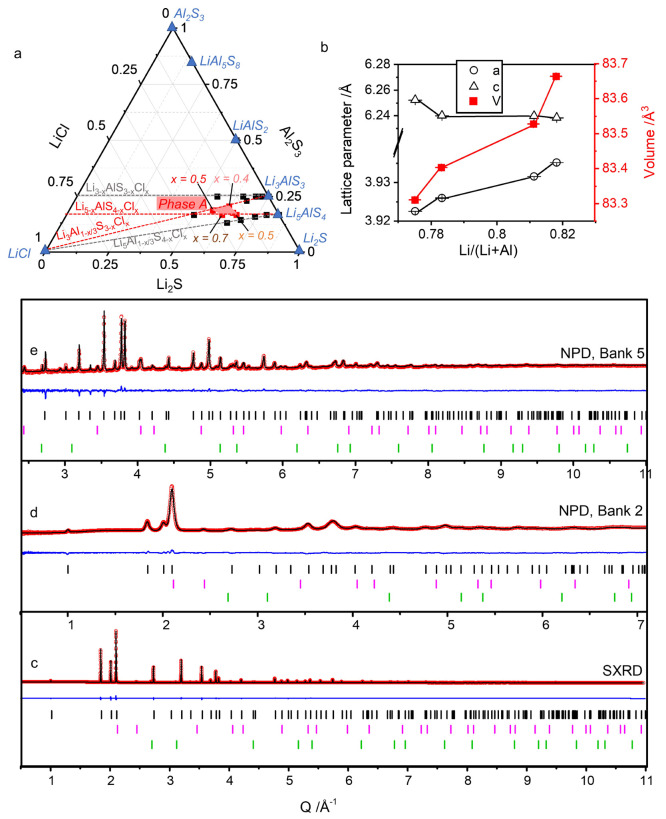
(a) Screening of S for Cl substitution in Li_3_AlS_3_ and Li_5_AlS_4_ following the four solid solution lines: Li_5–*x*_AlS_4–*x*_Cl_*x*_, Li_5_Al_1–*x*/3_S_4–*x*_Cl_*x*_, Li_3–*x*_AlS_3–*x*_Cl_*x*_, and Li_3_Al_1–*x*/3_S_3–*x*_Cl_*x*_. Known materials are represented by blue triangles, attempted compositions which resulted in mixed phase compounds by black squares, and compositions which lead to the new phase A with high purity by red squares. (b) Lattice parameters of phase A as a function of the amount of lithium. (c, d, e) Final Rietveld fit against (c) the SXRD data (λ = 0.825186 Å, Diamond, U.K.), and the NPD data from (d) Bank 2 (2θ = 25.990°) and (e) Bank 5 (2θ = 146.720°) of the Polaris instrument (ISIS, U.K.), with *I*_obs_ (red dots), *I*_calc_ (black line), *I*_obs_ – *I*_calc_ (blue line), and Bragg reflections (black tick marks for Li_4.3_AlS_3.3_Cl_0.7_, pink tick marks for LiCl (∼2 wt %), and green tick marks for Al (∼1 wt %).

Compositions were chosen along solid solution lines Li_5–*x*_AlS_4–*x*_Cl_*x*_, Li_5_Al_1–*x*/3_S_4–*x*_Cl_*x*_, Li_3–*x*_AlS_3–*x*_Cl_*x*_ and Li_3_Al_1–*x*/3_S_3–*x*_Cl_*x*_ (Figure 1a). For all compositions screened, reflections, which did not correspond to any known phases, appeared. These were attributed to a new phase (denominated phase A) which could then be isolated for four compositions along the solid solution lines Li_5–*x*_AlS_4–*x*_Cl_*x*_ (*x* = 0.5; 0.7) and Li_3_Al_1–*x*/3_S_3–*x*_Cl_*x*_ (*x* = 0.4; 0.5). Figure S1a (Supporting Information, SI) shows the XRD diagram of these four samples, and the slight shift of the peak positions with *Q* (Figure S1b) attests for a variation of their lattice parameters depending on the composition. Higher or lower *x* values and/or moving along the solid solution lines Li_5_Al_1–*x*/3_S_4–*x*_Cl_*x*_ and Li_3*–x*_AlS_3–*x*_Cl_*x*_ resulted in the formation of mixed phase compounds (example shown along the Li_3_Al_1–*x*/3_S_3–*x*_Cl_*x*_ solid solution line on Figure S2). The screening resulted in the delimitation of a small range of compositions for the formation of phase A with high purity (light red area in Figure 1a).

This new phase could be indexed to the *P*3̅*m*1 space group (Le Bail fit on Figure S1a for composition Li_4.3_AlS_3.3_Cl_0.7_, with *a* = 3.93161(3) Å and *c* = 6.23971(3) Å). All lattice parameters and cell volumes are reported in Table S1. The cell volume increases when the lithium to total cation ratio increases, consistent with the larger ionic radius of lithium (0.59 Å (IV) and 0.76 Å (VI)) compared to aluminum (0.53 Å (IV)), as shown in Figure 1b.^20^ Interestingly, this cell directly relates to that of Li_2_FeS_2_ (*P*3̅*m*1, *a* = 3.902(1) Å, *c* = 6.294(2) Å),^47^ whose structure can be viewed as a cation disordered analogue of Li_5_AlS_4_ and Li_3_AlS_3_ (Figure S3b–d). This seems promising for promoting ionic conduction and calls for further structural investigation.

An in-depth structural study, combining synchrotron X-ray diffraction (SXRD) and neutron powder diffraction (NPD), was performed on one of these compositions: Li_4.3_AlS_3.3_Cl_0.7_, corresponding to *x* = 0.7 in Li_5–*x*_AlS_4–*x*_Cl_*x*_. A small quantity of Al metal (coming from the starting material, Figure S4) and LiCl impurities were identified through preliminary Le Bail refinement, and these phases were added to the Rietveld refinement.

The Li_2_FeS_2_ (Li_4_Fe_2_S_4_ for compositional analogy) structure (Figure S3b) was used as a starting model where 1/6 of the S sites are occupied by Cl atoms (Wyckoff position 2*d*), Fe atoms are replaced by Al atoms with a site occupancy factor (*sof*) divided by 2 (Wyckoff position 2*d*, *sof* = 0.25) and the Li atoms distributed among the Al tetrahedral site (Li1, *sof* = 0.75) and the octahedral site (Li2, Wyckoff position 1*a*, *sof* = 0.5). This starting model showed a good fit to the SXRD data but fit poorly the NPD data at high *Q* (Figure S5a), pointing toward different Li site occupations, not well-determined using X-ray radiation. The Fourier difference map of Bank 5 of the NPD data highlighted a scattering density deficiency at the position (1/3, 2/3, 0.85) while showing an excess scattering density on the Al/Li1 site (Figure S5b). The latter was then split into two sites of different *z* positions. Eventually, positions, site occupancy factors, and anisotropic displacement parameters of all atoms were simultaneously refined against the combined SXRD and NPD data, showing a good fit to all data sets (Figure 1c–e and Figure S6) and yielding the final model. The outcome of the refinement is presented in Tables S2, S3, and S4. The overall composition refines to Li_4.32(1)_AlS_3.308(4)_Cl_0.71(2)_, close to the chemical composition measured by elemental analysis, Li_4.36(4)_Al_1.08(1)_S_3.30(3)_Cl_0.67(1)_, for which the slight deviation can be attributed to the presence of the Al metal and LiCl impurities (∼1 and 2 wt %, respectively, according to quantitative phase analysis with FullProf). The compound will be denoted Li_4.3_AlS_3.3_Cl_0.7_ hereafter for simplicity.

### 3.2 Structure Description

**Figure 2:**
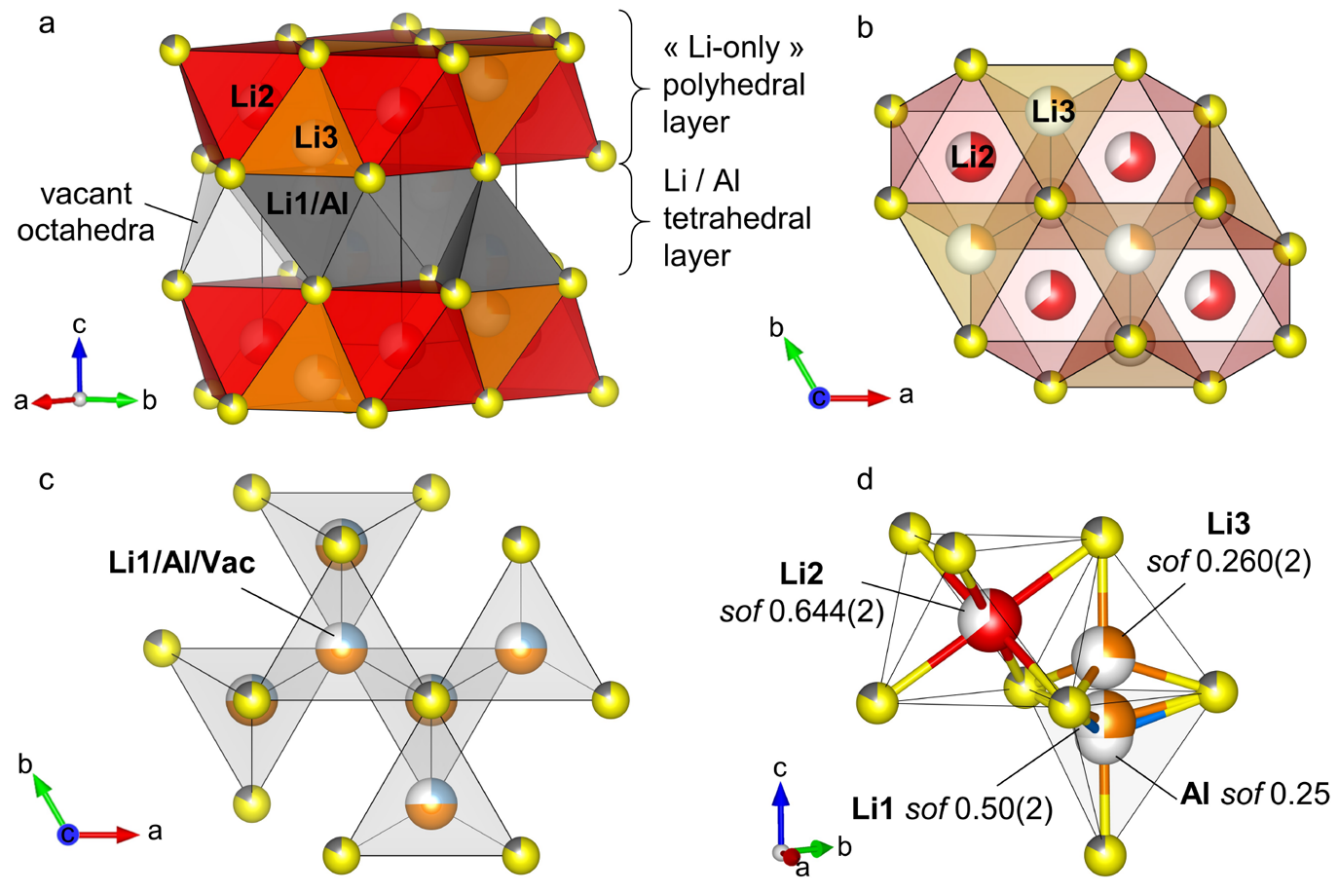
Crystal structure of Li_4.3_AlS_3.3_Cl_0.7_, with sulfur (yellow sphere) and chlorine (gray sphere) sharing the same site and arranged in a *hcp* type packing, Li atoms occupying octahedral (red) and tetrahedral (orange) interstices in the first layer, while Al (blue sphere) and remaining Li (orange sphere) atoms are randomly distributed among the tetrahedral interstices (gray) in the consecutive layers. (a) Layered view, (b) “Li-only” polyhedral layer in the (*ab*) plane, (c) tetrahedral layer in the (*ab*) plane, and (d) polyhedral coordination.

The structure consists of an anion sublattice in a *hcp* type packing arrangement. Sulfur and chlorine atoms are distributed randomly among the single anionic site in a S/Cl ratio of 0.827(3)/0.178(7), yielding a highly disordered structure. Cations occupy interstitial sites in between two anion slabs so that the structure exhibits two distinct layers alternating along the *c* axis (Figure 2a). In the first layer, lithium atoms partially occupy the octahedral (Li2, *sof* of 0.644(2)) and tetrahedral (Li3, *sof* of 0.260(2)) interstices (Figure 2b). In between the next two anion slabs, the two cations are randomly distributed among the T^+^ and T^–^ tetrahedral sites only, so that aluminum and lithium (Li1) are present in an Al/Li = 0.25/0.499(2) ratio (Figure 2c). This layer forms a “pure” tetrahedral layer.

In the “Li-only” polyhedral layer, Li2 octahedra are connected to six Li3 tetrahedra and six other Li2 octahedra of the layer via face and edge sharing, respectively (Figure 2b). The other two faces of the octahedra are part of the sulfur slab delimiting the layer and are connected to the fully vacant octahedral interstices of the tetrahedral layers above and below (Figure 2a).

Each T^+^ (T^–^) Al/Li1 tetrahedra is connected via edge sharing to the surrounding three T^–^ (T^+^) tetrahedra of the layer (Figure 2c). It is connected to the consecutive layers by sharing its three remaining edges (from the base) and one corner (the apex), with the Li2 octahedra from the above and below polyhedral layer, respectively (Figure 2a). The face of the base and the apex of each of these tetrahedra are also shared with that of the below and above Li3 tetrahedra, respectively (Figure 2a,d). These two face-shared tetrahedra form a unit, in which the hypothetical Al–Li3 and Li1–Li3 distances are very small (*d*_Al–Li3_ = 1.543(13) Å and *d*_Li1–Li3_ = 1.274(14) Å, Figure 2d), rendering their mutual occupation within the same unit very unlikely. The combined site occupancy factor of the unit refines to 1.009(4), very close to a full occupancy, suggesting that the unit hosts exactly one atom, either in the Li3 or in the Li1/Al position. As such, accessible disordered vacancies are distributed among Li2 octahedral sites only (*sof*_Li2_ = 0.644(2)).

The random distribution of S and Cl atoms leads to the presence of different heteroanionic polyhedra AlS_4–*m*_Cl_*m*_, LiS_4–*m*_Cl_*m*_ and LiS_6–*n*_Cl_*n*_ (0 ≤ *m* ≤ 4; 0 ≤ *n* ≤ 6) distributed in a disordered manner within the material.

**Figure 3:**
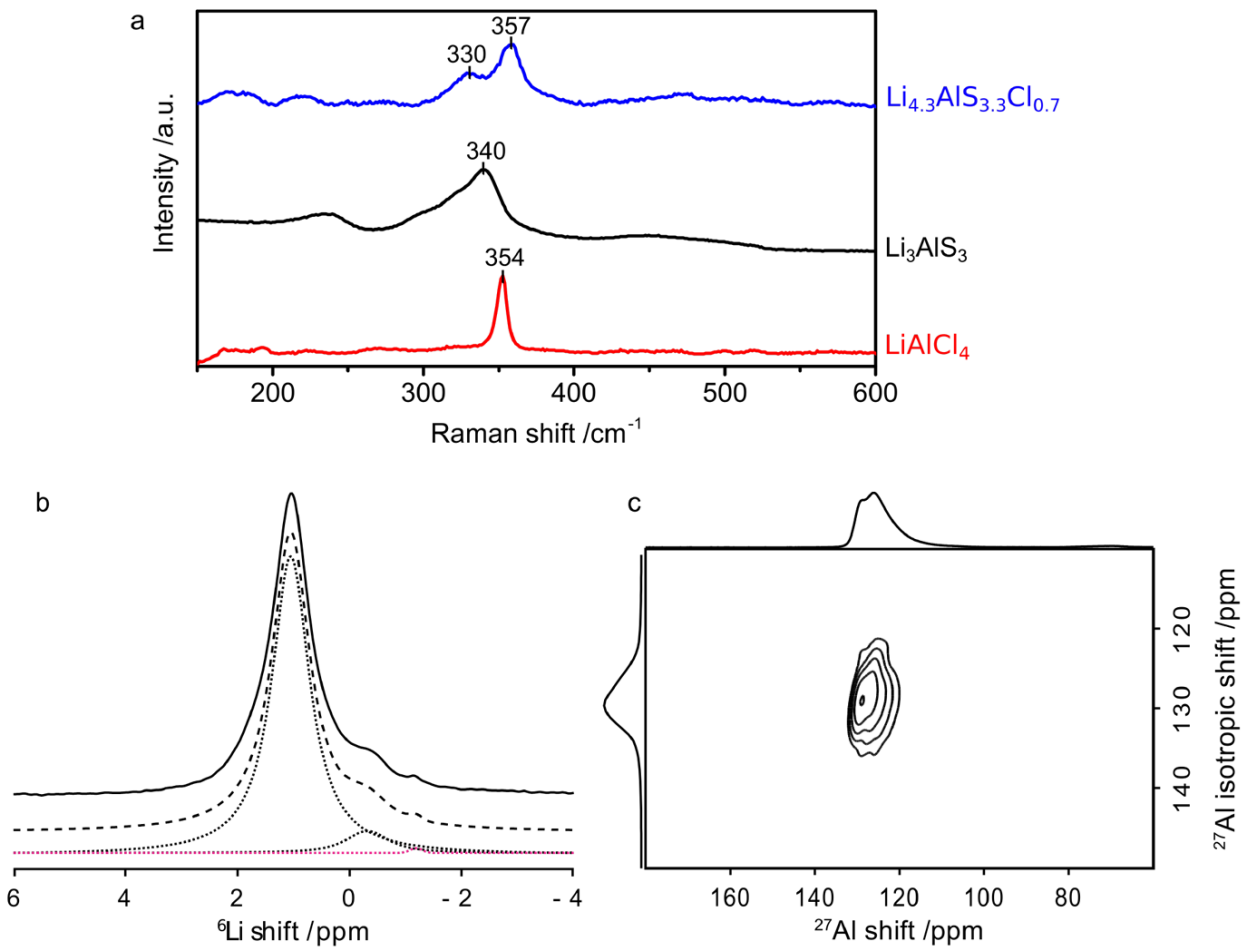
(a) Raman spectra of Li_4.3_AlS_3.3_Cl_0.7_, Li_3_AlS_3_, and reference material LiAlCl_4_. (b) ^6^Li MAS spectrum of Li_4.3_AlS_3.3_Cl_0.7_. The experimental spectrum (full line), total fit (dashed line), and spectral deconvolution (dotted lines) are also shown. (c) ^27^Al MQMAS spectrum of Li_4.3_AlS_3.3_Cl_0.7_. The spectrum on the top is the ^27^Al MAS NMR spectrum, while the one on the left is the isotropic ^27^Al spectrum free of anisotropic broadening.

Raman spectroscopy was carried out on the title compound Li_4.3_AlS_3.3_Cl_0.7_ as well as on two reference materials Li_3_AlS_3_ and LiAlCl_4_. The Raman spectra of Li_4.3_AlS_3.3_Cl_0.7_ show two broad bands centered at 330 and 357 cm^–1^ (Figure 3a). These correspond to the stretching vibration of the Li–*X* and Al–*X* bonds in the tetrahedral symmetry (*X* = S, Cl).^48−50^ The more intense and higher frequency band can be attributed to Li–S vibrations while the less intense and lower frequency band to the vibration of the heavier Al atom. Indeed, a calculation of the vibration frequency considering a simple harmonic oscillator gives averaged vibration frequencies of 315(3) cm^–1^ and 197(1) cm^–1^ for Li–S and Al–S bonds, respectively (Table S5). In comparison, Li_3_AlS_3_ shows an even broader asymmetric band between 300 and 350 cm^–1^, with a maximum at 340 cm^–1^. This also encompasses the stretching vibrations of Li–S and Al–S bonds. The broadness of the band reflects the low symmetry of the material, which comprises a range of Li–S distances.^31^ The shift of the band maximum toward higher frequency in Li_4.3_AlS_3.3_Cl_0.7_ (357 cm^–1^) compared to Li_3_AlS_3_ (340 cm^–1^) could be explained by the lower averaged Li–S distance in Li_4.3_AlS_3.3_Cl_0.7_ (*d*_Li–_*X*,av = 2.391(26) Å) compared to Li_3_AlS_3_ (*d*_Li–S,av_ = 2.473(92) Å). Indeed, the averaged calculated frequency using the simple harmonic oscillator approach gives a value of 300(1) cm^–1^ for the Li–S bond in Li_3_AlS_3_. Moreover, the differences between the averaged calculated frequencies of the Li–*X* and Al–*X* vibrations are larger in Li_4.3_AlS_3.3_Cl_0.7_ (Δν_Li–_*X*/Al–*X* = 118(4) cm^–1^) than in Li_3_AlS_3_ (Δν_Li–_*X*/Al–*X* = 75(2) cm^–1^, Table S5). This suggests that the Al–S vibration band is merged with that of Li–S in Li_3_AlS_3_, which can explain why a second resolved band is not observed in this material on the contrary to Li_4.3_AlS_3.3_Cl_0.7_. As a comparison, the Raman spectra of the reference sample LiAlCl_4_ was recorded and shows a sharp resonance at 354 cm^–1^ corresponding to the symmetric stretching vibration of the Al–Cl bond in the *T*_d_ symmetry. The Al–Cl distance in LiAlCl_4_ (*d*_Al–Cl,av_ (LiAlCl_4_) = 2.135 Å) is lower than in Li_4.3_AlS_3.3_Cl_0.7_ (*d*_Al–_*X*,av (Li_4.3_AlS_3.3_Cl_0.7_) = 2.388 Å), suggesting that Al–Cl vibration bands in the Li_4.3_AlS_3.3_Cl_0.7_ spectra will appear at lower frequency, within the same region as the Li–S and Al–S vibration bands.

Multinuclear ^6^Li and ^27^Al NMR spectra were recorded to further support the structural refinement. The ^6^Li MAS NMR spectrum (Figure 3b) displays an intense resonance at 1 ppm assigned to tetrahedral and octahedral sites from the Li_4.3_AlS_3.3_Cl_0.7_ phase and a small peak at ∼−0.3 ppm, which could potentially be attributed to small amounts of octahedral lithium sites from the ordered Li_3_AlS_3_ phase.^31^ A smaller peak at −1.1 ppm is also visible and corresponds to solid LiCl^51^ (observed in XRD). The main signal is narrow (50 Hz) at room temperature and suggests the presence of a motionally averaged NMR signal arising from fast Li^+^ hops and preventing the spectral resolution of Li sites with various coordination numbers. The ^27^Al MAS NMR spectrum (Figure 3c) shows the presence of two asymmetrically broadened and overlapping peaks around 125 ppm which are assigned to Al tetrahedra based on the shift value (note that the quadrupolar induced shift^52^ is likely smaller than 5 ppm at this magnetic field). Less intense resonances at 70 and 16 ppm (Figure S7) are assigned to a small amount of more highly coordinated Al. Most importantly, the second-order quadrupolar line shape observed for an AlS_4_ tetrahedron in the parent Li_3_AlS_3_^31^ is not observed and further supports that Li_4.3_AlS_3.3_Cl_0.7_ cannot be described by distinct AlCl_4_ and AlS_4_ tetrahedra but by a random distribution of the S and Cl atoms. The asymmetrically broadened lines arise from second-order quadrupolar interaction coming from deviation from the perfect tetrahedral site symmetry while the low frequency tail is cause by a distribution of quadrupolar couplings stemming from local structural disorder. An attempt to resolve the main resonances using a ^27^Al MQMAS NMR experiment only yields the typical 2D line shape from distribution of quadrupolar couplings, and no improvement in the resolution of the corresponding ^27^Al isotropic spectrum is observed.

As expected from a simple S to Cl substitution mechanism, the backbone of the parent material structures, Li_5_AlS_4_ and Li_3_AlS_3_, is maintained for the substituted phase Li_4.3_AlS_3.3_Cl_0.7_. The three structures present an *hcp* type packing of anions (either S^2–^ only or a random distribution S^2–^/Cl^–^) and alternating tetrahedral Al/Li layers with Li-only polyhedral layers. Indeed, the similarity in the ionic radius of Cl^–^ (1.81 Å) and S^2–^ (1.84 Å) is favorable to site sharing and therefore helps to maintain structural integrity (Figure S3a,c,d).

However, the cation arrangement within each layer is considerably different in the pure sulfide and in the sulfide–chloride phases, so that Li_5_AlS_4_ and Li_3_AlS_3_ are superstructures of Li_4.3_AlS_3.3_Cl_0.7_. In Li_5_AlS_4_, Al and Li are ordered among the tetrahedral interstices of the tetrahedral layer in a 1:3 arrangement, and the octahedral sites of the “Li-only” layer are fully occupied. In Li_3_AlS_3_, in the tetrahedral layer, Al, Li, and vacancies are ordered in a 1:1:1 arrangement, and 2/3 of the octahedral interstices are occupied in the Li-only layer, so that this structure presents a high proportion of ordered vacancies in both the tetrahedral and Li-only layer. The presence of Cl in the structure promotes the formation of a higher symmetry phase, with a high degree of site disorder, as well as the presence of disordered vacancies, which is expected to have a major impact on the Li mobility.

### 3.3 Phase Stability Calculation

Such disorder is absent in the pure sulfide compositionally related phases Li_5_AlS_4_ and Li_3_AlS_3_ which calls for an understanding of the effect of Cl^–^ substitution on the formation of disorder in this structure type. The Gibb’s free energy of Li_5_AlS_4_, Li_3_AlS_3_, and Li_4.3_AlS_3.3_Cl_0.7_ materials in their hypothetical ordered (*G*_o_) and disordered (*G*_d_) structure was calculated (*cf.*Experimental Section). The Gibbs free energy deviation Δ*G* = *G*_o_ – *G*_d_ indicates whether the ordered (if Δ*G* > 0) or disordered (if Δ*G* < 0) structure is more energetically favorable at a given temperature. In Li_5_AlS_4_ and Li_3_AlS_3_, Δ*G* is negative at *T* > 1190 °C and *T* > 688 °C, respectively, whereas for Li_4.3_AlS_3.3_Cl_0.7_, Δ*G* is negative at *T* > 250 °C. This decrease in temperature is due to the contribution of configurational entropy (*cf. G*(*T*) plots in the SI, Figure S8). In these calculations, the contribution from the vibrational entropy was not taken into account, which impedes direct comparison of theoretically obtained free energy values for these compositions. However, the significant reduction in transition temperature, close to room temperature, indicates that, via increased mixing entropy, Cl doping facilitates thermodynamic stabilization.

### 3.4 Lithium Ionic Conductivity

**Figure 4:**
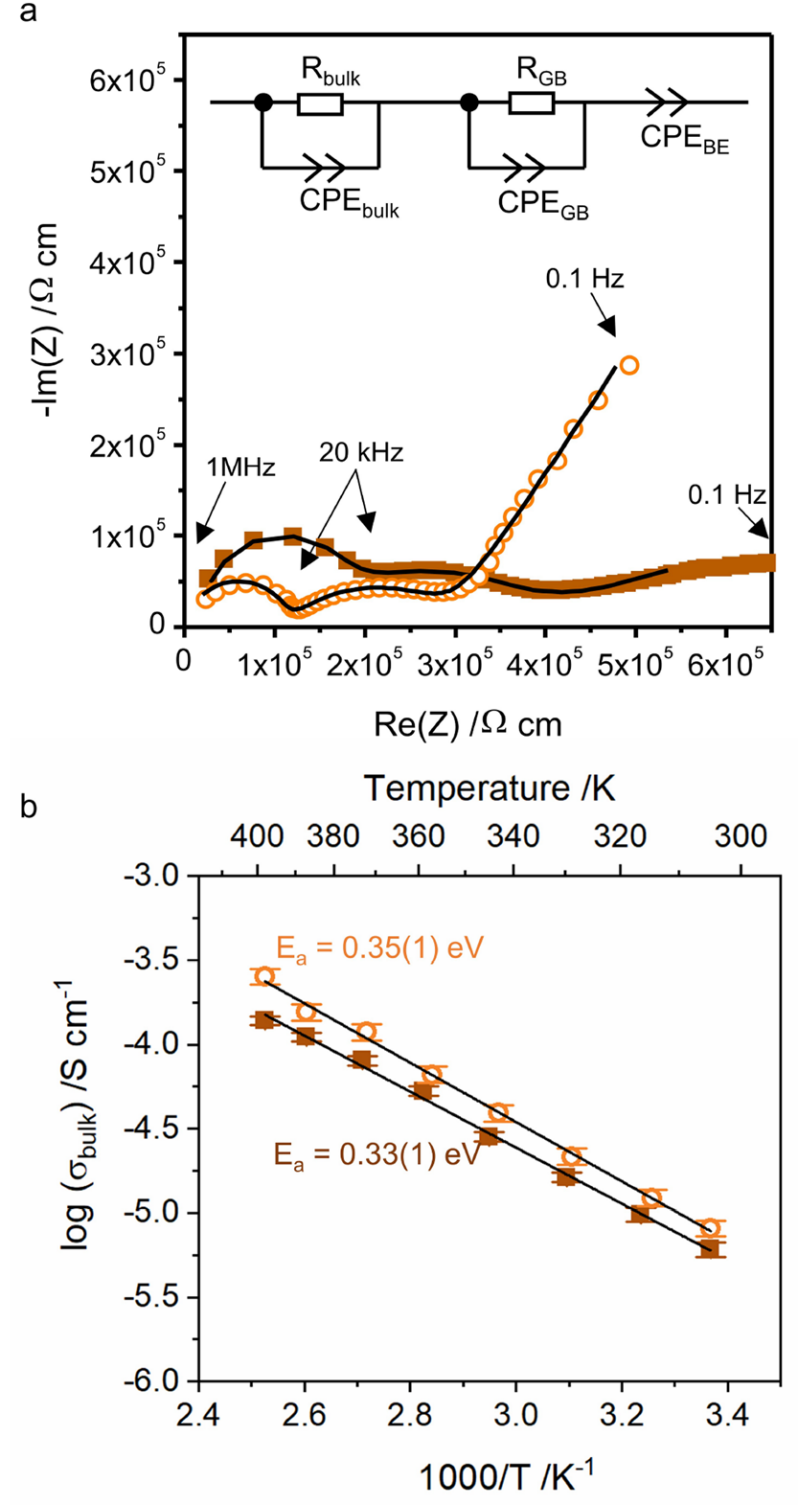
(a) Room temperature (303 K) Nyquist plot of Li_4.3_AlS_3.3_Cl_0.7_ (filled brown squares) and Li_4.5_AlS_3.5_Cl_0.5_ (empty orange circles) and their fit using the equivalent circuit in inset (black line), showing the two contributions to the conductivity, and (b) Arrhenius plot of the bulk conductivity of the two samples measured by AC impedance and the linear fit using Arrhenius law (black line). CPE stands for constant phase element, R for resistance, GB for grain boundary, and BE for blocking electrode.

The lithium ionic conductivity was measured by AC impedance spectroscopy on a sintered pellet. The room temperature Nyquist plots of Li_4.5_AlS_3.5_Cl_0.5_ and Li_4.3_AlS_3.3_Cl_0.7_ are presented on Figure 4a. The presence of the two semicircles is characteristic of the deconvolution of two diffusion phenomena occurring on different time scales, whereas the low frequency region corresponds to the response at the electrode interface. Plots were fitted using a typical equivalent circuit presented in the inset of Figure 4a to take into account these three contributions and obtain the conductivities. The first two components consist of a resistance in parallel with a Constant Phase Element (CPE, a modified capacitor taking into account inhomogeneities in the sample). The electrode response was modeled using a CPE. Result of the fits are presented in the SI (Table S6).

For the Li_4.5_AlS_3.5_Cl_0.5_ sample, the values of the capacitance for the first and second contributions are 8(4) × 10^–12^ F and 6(3) × 10^–10^ F. These values are characteristic of the response from ion diffusion in the bulk and across the grain boundary, respectively.^53^ The high porosity of the sample is reflected in the low value of *n*_GB_ for the grain boundary contribution (Table S6), giving rise to a suppressed semicircle. The high frequency intercepts of both semicircles give direct values of the bulk and grain boundary resistance. The room temperature bulk and total conductivity (bulk and grain boundary contributions added) are σ_bulk_(303 K) = 8.1(9) × 10^–6^ S·cm^–1^ and σ_tot_(303 K) = 3.7(4) × 10^–6^ S·cm^–1^, respectively. The steep low frequency tail is characteristic of an ion blocking electrode. A polarization measurement was performed to estimate the electronic contribution to the total conductivity, which revealed to be negligible compared to the ionic contribution (σ_e_= 0.010(2)% × σ_tot_, Figure S9). AC impedance was measured over the temperature range 24–125 °C, and each Nyquist plot was fitted using the same equivalent circuit. σ_bulk_ was extracted at each temperature point and shown to follow the Arrhenius law, with activation energy, *E*_a_, of 0.35(1) eV (Figure 4b).

The impedance spectra of the Li_4.3_AlS_3.3_Cl_0.7_ sample and its evolution with temperature are similar to those of the Li_4.5_AlS_3.5_Cl_0.5_ sample with values of capacitances of 1.0(4) × 10^–11^ F and 2.1(3) × 10^–8^ F for the first and second contributions, respectively. Values of conductivities were σ_bulk_(303 K) = 6.1(6) × 10^–6^ S·cm^–1^ and σ_tot_(303 K) = 2.5(2) × 10^–6^ S·cm^–1^, and the activation energy was 0.33(1) eV (Figure 4b). The electrode response for Li_4.3_AlS_3.3_Cl_0.7_ shows a weak slope, which does not fully correspond to a blocking electrode behavior, and could be attributed to a residual electronic conductivity at the interface due to the reduced sintering treatment (*cf.*Experimental Section). Indeed, the impedance spectra of the sample before sintering shows the presence of the blocking electrode attesting the pure ionic conductor behavior of the material before sintering and pointing out the need for optimization of the sintering procedure for further use of the material (Figure S10).

Compared to the pure sulfide materials Li_5_AlS_4_ and Li_3_AlS_3_, which showed room temperature conductivities of σ_tot_ = 9.7 × 10^–9^ S·cm^–146^ and σ_bulk_ = 1.3(1) × 10^–8^ S·cm^–1^,^31^ respectively, the Li bulk mobility is increased by almost 3 orders of magnitude in the mixed chloride–sulfide phases. Concomitantly, the activation energy decreases by 30 to 40%, with *E*_a_ = 0.61 eV for Li_5_AlS_4_^46^ and *E*_a_ = 0.48 eV for Li_3_AlS_3_.^31^

Likewise, Leube et al. recently reported a major conductivity increase in this structure type, thanks to the introduction of a 4+ charged cation in the pure sulfide phase Li_5_AlS_4_.^37^ The bulk conductivity of Li_4.4_Al_0.4_Ge_0.6_S_4_ in particular is at least as high as the reported total conductivity σ_tot_ = 4.3(3) × 10^–5^ S·cm^–1^. The conductivity increase compared to Li_5_AlS_4_ or Li_3_AlS_3_ was attributed to the presence of disordered vacancies among the lithium sites while maintaining the highly ordered anion S^2–^ sublattice and the site differentiation between Li^+^ and non-mobile cations.^37^ The conductivity of Li_4–*x*_AlS_3–*x*_Cl_*x*_ (*x* = 0.5 ; 0.7) remains in the same conductivity range as that of the Li_4.4_*M*_0.4_*M*′_0.6_S_4_ (*M* = Al, Ga; *M*′ = Ge, Sn) compounds, while being 1 order of magnitude lower than that of the best material in the series, Li_4.4_Al_0.4_Ge_0.6_S_4_.

In order to find an explanation for the observed differences and similarities in the Li mobility of these materials, the study of the diffusion mechanism was undertaken.

### 3.5 Lithium Diffusion Pathways

In order to visualize conduction pathways, ab initio molecular dynamics (AIMD) was conducted on the highest symmetry and most stable structure generated from a (2*a*, 2*b*, 2*c*) supercell of the Li_4.3_Al_3.3_Cl_0.7_ experimental structure (*cf.*Experimental Section). The structure is shown in Figure S11 and presents the overall composition Li_13_Al_3_S_10_Cl_2_ (Li_4.3_AlS_3.3_Cl_0.7_). As the experimental structure does, it consists of tetrahedral layers containing Al and Li, alternating with “Li-only” layers. One “Li-only” layer (the one in the center of the cell) shows fully occupied octahedral sites and fully vacant tetrahedral sites. It is bounded by pure sulfide layers above and below it. The other “Li-only” layer (the one at the bottom/top) has two octahedral sites and four tetrahedral sites occupied, leaving four octahedral sites vacant, and is bounded on one side by the chloride ions in the structure.

**Figure 5:**
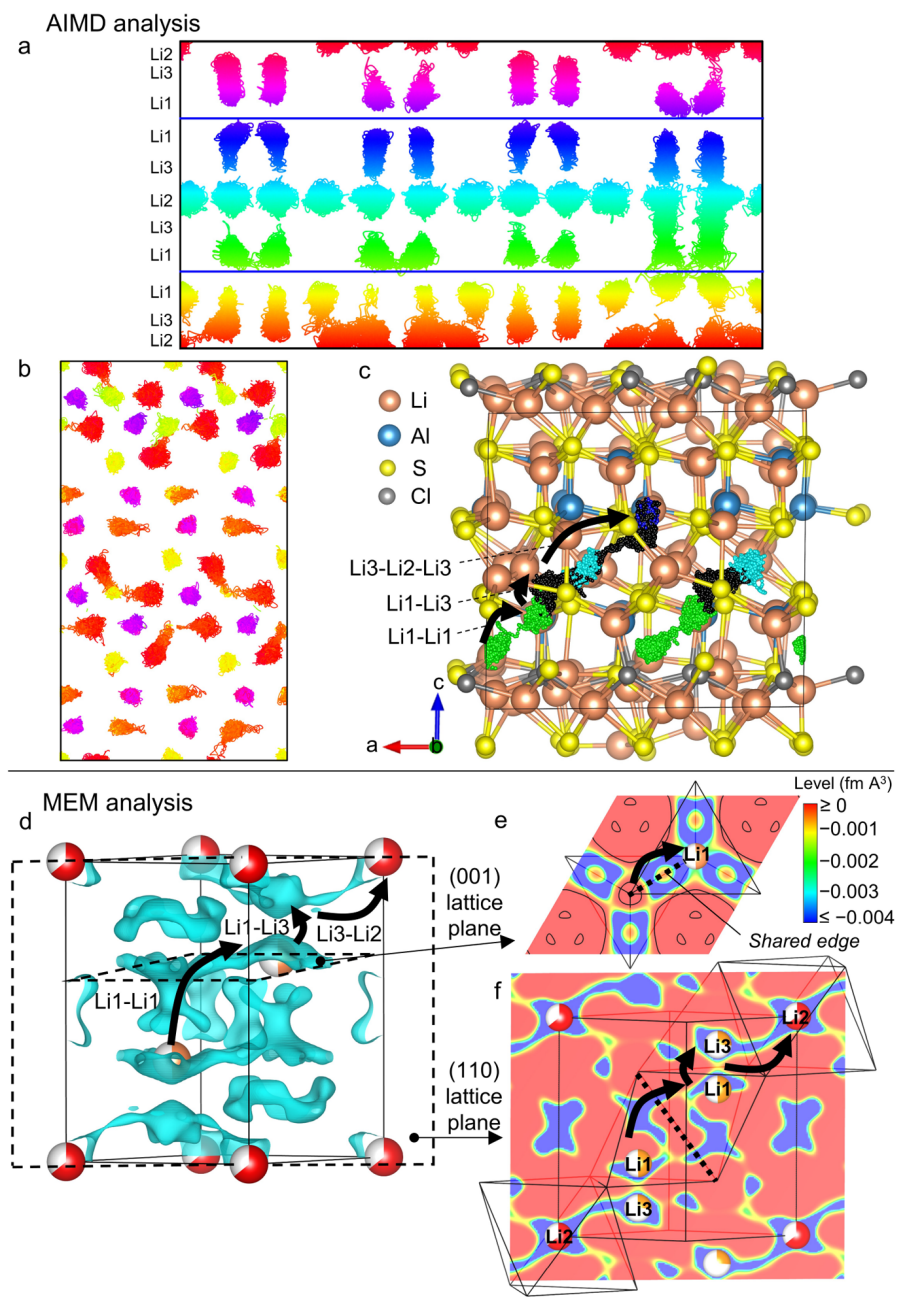
Visualization of Li diffusion pathways using AIMD (a, b, c) and MEM (d, e, f). (a and b) Positions of Li ions within the Li_104_Al_24_S_80_Cl_16_ supercell over a 100 ps AIMD trajectory. Atoms are colored according to their position along the *c* axis. (a) View along the *a* axis. The cell is split into two halves using the heights shown with blue lines. (b) Cell viewed down the *c* axis showing the half of the cell centered on the octahedral layer at the bottom/top of the cell. (c) View of five Li atoms in the structure throughout their AIMD trajectory. The mobile Li atoms are colored according to which site they belong, as in (a): green and blue for Li1, cyan for Li2, an exception is made for Li3, colored in black, in order to distinguish it more clearly. The other atoms are frozen in the positions they have at the start of the AIMD production run. (d, e, f) Nuclear density reconstructed by the maximum entropy method using Bank 5 of the NPD data of Li_4.3_AlS_3.3_Cl_0.7_ and highlighted potential diffusion pathways, matching those observed with AIMD on (b). (d) 3D Isosurface of the negative nuclear density within the cell (level = −0.004 fm A^3^), (e) 2D nuclear density map in the (001) plane passing through Li1, and (f) 2D nuclear density map in the (110) plane passing through Li2, Li3, and Li1.

A larger supercell was created with composition Li_104_Al_24_S_80_Cl_16_ and was used for a 100 ps *ab initio* MD run at 400 K. The trajectories of the Li ions throughout the production run are shown in Figure 5a,b. Figure 5b shows that some site-to-site Li hopping occurs within the “Li-only layer” from one octahedral site to another, through the intermediate tetrahedral site (yellow) with which it shares a common face. This corresponds to Li2–Li3–Li2 hops (O–T–O).

Moreover, some site-to-site hopping is observed across the tetrahedral layer (Figure 5a, bottom right, orange to blue) involving Li3–Li1 as well as Li1–Li1 hops and suggesting that the transport may not be solely two-dimensional. The Li1–Li1 hops (green to yellow on Figure 5a) seem to happen much less frequently than the Li2–Li3 and Li1–Li3 hops, which indicates higher activation energy involved in the tetrahedral to tetrahedral jumps. Interestingly, this observation strongly differs with the Li trajectories obtained in the related structure Li_4.4_Al_0.4_Ge_0.6_S_4_, for which 2D diffusion within the octahedral layer only was determined through a combined NMR and AIMD analysis.^37^ As explained in Section 3.2, Li_4.4_Al_0.4_Ge_0.6_S_4_ presents a more ordered structure with differentiation of the Ge/Al and Li tetrahedral sites and the presence of ordered octahedral vacancies in the octahedral layer.

The influence of disorder on the conductivity dimensionality was further examined with AIMD using the structure of Li_4.4_Al_0.4_Ge_0.6_S_4_ as a starting point (Figure S3e) while keeping the Li_4.3_AlS_3.3_Cl_0.7_ composition. The ordered octahedral vacant sites and Li/Al ordering within the tetrahedral layer were kept from Li_4.4_Al_0.4_Ge_0.6_S_4_ with the S/Cl and Li site disorder introduced to match Li_4.3_AlS_3.3_Cl_0.7_. The optimized and lowest energy structure is presented on Figure S12. A supercell with composition Li_104_Al_24_S_80_Cl_16_ was constructed and used for a 100 ps AIMD production run at 400 K. The trajectory of Li ions during the run is shown in Figure S13. The octahedral sites (purple in Figure S13b and light green in Figure S13c) do not show any site-to-site hopping. Some isolated hopping events do appear to happen from tetrahedral sites into the vacant octahedral sites and back (e.g., pink Li at top center of Figure S13b). However, on the contrary to Li1–Li1 hops observed in the model with the experimental structure, no hopping of tetrahedral Li to tetrahedral Li within the tetrahedral layer can be seen. This comparison highlights the importance of a higher degree of atomic disorder for accessing more diverse hopping pathways.

Further visualization of the 3-dimentional Li diffusion pathways in the disordered Li_4.3_AlS_3.3_Cl_0.7_ was performed by tracking the position of five different selected Li atoms over a 100 ps period, showing their movement throughout the trajectory (Figure 5c). The Li atoms are colored according to which site they belong. Diffusion within and across the tetrahedral layer is clearly identified with Li1–Li1 hops through their shared edge (green) concomitantly with Li1–Li3 exchange across the shared tetrahedral face (green–black). Diffusion within the “Li-only” layer is observed with Li3–Li2–Li3 hops (black–cyan–black) occurring via the shared octahedral–tetrahedral faces. As stated in the structure description section, the Li1–Li3 unit can only host one ion at the same time. Therefore, the migration mechanism can only happen if the Li3 position is being vacated while another Li^+^ ion moves to the Li1/Al site of the same unit. The synchronicity of these three hopping events is indeed observed within a period of less than 7 ps (*cf.*Figure S14). This leads to a knock-on mechanism responsible for concerted migration of Li within the structure. This mechanism is different from the classical direct hopping mechanism, where isolated Li hop events happen through empty interstices, and has been shown to be responsible for fast ionic conductivity in different types of materials.^54^

Experimental evidence for Li diffusion pathway was obtained through the analysis of the nuclear density maps derived from neutron diffraction data using the maximum entropy method (MEM, *cf.*Experimental Section). Figure 5d shows the nuclear density isosurface map within the Li_4.3_AlS_3.3_Cl_0.7_ cell. Visualization of negative levels is performed, enabling Li positions to be distinguished (^7^Li scattering length is negative: *b*_Li_ = −2.22 fm) and hopping pathways to be identified. These are marked with arrows in Figure 5d–f and are in perfect accordance with pathways obtained from AIMD: Li1–Li1 hops through their shared edge (dotted line on Figure 5e,f), Li1–Li3 hops through the shared face, and Li3–Li2 hops through the common tetrahedral–octahedral face. The comparison between AIMD and MEM analysis applied to the same material is rarely performed in the literature. The theoretical AIMD method models the dynamic evolution of a local environment, thereby giving a direct the trajectory of atoms, and the MEM analysis gives an average nuclear density within a crystal cell, thereby showing places where Li atoms should be. Here, the exact correspondence between results shows that it is possible to link experimental data of the average structure to a detailed understanding of local motion.

## 4 Discussion

The substitution of sulfide for chloride anions into the pure sulfide materials Li_3_AlS_3_ and Li_5_AlS_4_ leads to the formation of a new phase, showing major differences in its structure and Li conductivity properties.

First, while the substitution maintains the two layer type arrangement (tetrahedral Li/Al layers alternating with “Li-only” layers, in between anion slabs packed in a *hcp* manner), it generates a high degree of atomic disorder. This disorder is observed both in the anionic sublattice (through a random occupancy of the anion site by S^2–^ and Cl^–^) and within the cationic sublattice with a random occupancy of Al and Li in the tetrahedral sites, as well as the partial occupancy of the octahedral sites, leading to the presence of disordered vacancies to the amount of 35.6(2)%. The observation that anion doping leads to a disordered structure has been reported in the literature in various type of compounds,^10−15^ although the origin for this structural behavior remains unclear. By calculating the stabilization temperature of disordered and ordered structures in both chloride-substituted and non-substituted materials, we prove that the structure is thermodynamically stabilized by the presence of Cl. Indeed, it cannot be explained by kinetic consideration on their own, such as differences in the reaction kinetics of the starting materials. Thermodynamic stabilization of the disordered structure could be explained by the increase in configurational entropy brought by the insertion of a second chemical element on the same atomic site or by the randomization of interstitial site geometries.

Second, Cl substituted materials show major differences in the conductivity properties compared to the related pure sulfide materials. The conductivity values are increased by a factor 10^3^ compared to the ordered sulfide phases Li_3_AlS_3_ and Li_5_AlS_4_^31,46^ but are of the same order to magnitude as those of Li_4.4_*M*_0.4_*M*′_0.6_S_4_ (*M* = Al, Ga; *M*′ = Ge, Sn).^37^ The latter presents some Li site vacancies while maintaining an ordered *M*/*M*′ *vs*. Li site arrangement and the presence of ordered octahedral vacancies. This increase in conductivity is attributed to the presence of disordered vacancies in both types of materials, absent in the ternary compounds.

Further insight in the limiting transport mechanism is given through the comparison of diffusion pathways in fully disordered Li_4.3_AlS_3.3_Cl_0.7_ (modeled using the experimental structure) and in the partially disordered Li_4.3_AlS_3.3_Cl_0.7_ (modeled using the Li_4.4_Al_0.4_Ge_0.6_S_4_ structure). It suggests that introducing more disorder in Li_4.3_AlS_3.3_Cl_0.7_, *i.e.*, Li/Al site disorder in the tetrahedral layer and fully disordered octahedral vacancies, enables 3D hopping pathways to be accessed, favorable for enhanced Li conductivity. This effect cannot be attributed to the presence of Cl only nor to the modification in the local geometry of atoms, as these two effects are kept unchanged in both models. Rather, this could find its origin in the increased entropy of the material, which has a fundamental effect on the ion dynamics. From a certain level of disorder, migration involves several ions at the same time: the movement of one ion automatically results in that of one or more others in response.^55,56^ The activation of this concerted ion migration mechanism is therefore sought to increase ionic conductivity.^57^ One explanation for obtaining this type of mechanism is the occupation, by the mobile ion, of both a high-energy site and a more stable low energy site. This has the effect of reducing the potential barrier as the ion jumps from the high energy site to the low energy site and activates the concerted jump mechanism.^54^ This mechanism is observed with AIMD in Li_4.3_AlS_3.3_Cl_0.7_ and is schematized on Figure S15.

The mediocre conductivity in Li_4.3_AlS_3.3_Cl_0.7_ compared to Li_4.4_Al_0.4_Ge_0.6_S_4_ could be explained by the presence of Cl^–^ anions, with higher electronegativity and hence lower polarizability than S^2–^, thereby decreasing the overall Li bond covalency and increasing overall activation energy, thereby counterbalancing the positive effect of 3D diffusion pathways.

In order to further increase conductivity in this structure type, one must take advantage of both effects: 3D diffusivity and low activation energy. For instance, heterovalent doping on either the anionic (with higher polarizable anions such as Br^–^, I^–^) or the cationic sites could be interesting to increase Li bond covalency, enlarge the unit cell volume, and open a wider bottleneck, while maintaining the high degree of atomic disorder, necessary for 3D conductivity.

## 5 Conclusion

A novel sulfide chloride phase was identified in the Li–Al–S–Cl phase diagram, with composition spanning Li_5–*y*_Al_1+(*y*–*x*)/3_S_4–*x*_Cl_*x*_ (*x* = 0.5–0.7; *y* = 0.5–1). Its structure resembles that of Li_2_FeS_2_ and can be described as a disordered analogue to that of the parent sulfide phases Li_3_AlS_3_ and Li_5_AlS_4_. The thermodynamic stabilization of this high symmetry phase and the presence of large atomic disorder was facilitated thanks to the introduction of a chloride anion on the sulfur site, as revealed by phase stability calculations. In depth crystallographic characterization was performed on Li_4.3_AlS_3.3_Cl_0.7_ (*x* = 0.7; *y* = 0.7) by means of combined high-resolution X-ray and neutron diffraction together with NMR spectroscopy. Neutron diffraction in particular enabled major differences to be revealed in the lithium site position and occupation compared to the sulfide phases, with the localization of disordered vacancies among the octahedral sites only as well as the splitting of tetrahedral lithium atoms. A combined experimental–theoretical approach revealed the major impact of these defects on the conductivity properties, as both take part in the main diffusion pathway, in turns leading to an increase of 3 orders of magnitude in the Li conductivity. Remarkably, AIMD and MEM evidenced exactly the same Li hopping paths. This directly supports the relevance of using MEM associated with neutron diffraction to determine diffusion pathways from experimental data. Moreover, a correlation is made between high atomic disorder and the access to a 3D conductivity pathway, which was revealed for the first time in this structure type. By analyzing the strong impact of chlorine for sulfur substitution, we highlight a path for the exploration of new promising mixed anion Li electrolyte materials.

## Supporting Information Available

Structural information on the four “phase A” compositions (SRXD patterns, lattice parameters, and cell volume), laboratory XRD patterns of additional impure sample within the Li–Al–S-Cl phase field, Comparison of the crystal structures of Li_4.3_AlS_3.3_Cl_0.7_, Li_2_FeS_2_, Li_5_AlS_4_, Li_3_AlS_3_ and Li_4.4_Al_0.4_Ge_0.6_S_4.4_, Preliminary Rietveld refinement and Fourier difference map of Li_4.3_AlS_3.3_Cl_0.7_, final Rietveld fit against the NPD data from Bank 3 and Bank 4, summary of the outcome of the refinement, crystal structure of Li_4.3_AlS_3.3_Cl_0.7_, Δ*G* vs temperature plots, structural models used for AIMD, and AIMD trajectories in the hypothetical partially ordered structure of Li_4.3_AlS_3.3_Cl_0.7_ (PDF)
Associated structural information on Li_4.3_AlS_3.3_Cl_0.7_, also available from the Cambridge Structural Database (CSD deposition number 2050422) (CIF)

## Supplementary Material

cm1c02751_si_001.pdf

cm1c02751_si_002.cif
