# Supplementary material for: Li4.3AlS3.3Cl0.7: A Sulfide–Chloride Lithium Ion Conductor with Highly Disordered Structure and Increased Conductivity
Source: Chem Mater. 2021 Nov 10;33(22):8733–44. doi: 10.1021/acs.chemmater.1c02751 (PMC8613839; doi:10.1021/acs.chemmater.1c02751)
Supplement: Supplementary file 1 — cm1c02751_si_001.pdf [file cm1c02751_si_001.pdf]

$Li_{4.3}AlS_{3.3}Cl_{0.7}$: A Sulfide-Chloride Lithium Ion Conductor with Highly Disordered Structure and Increased Conductivity

Jacinthe Gamon[1], Matthew S. Dyer[1,3], Benjamin B. Duff[1,2], Andrij Vasylenko[1], Luke M. Daniels[1], Marco Zanella [1], Michael W. Gaultois[1,3], Frédéric Blanc[1,2,3], John B. Claridge[1,3] and Matthew J. Rosseinsky[1,3]*

[1]Department of Chemistry, University of Liverpool, Crown Street, L69 7ZD Liverpool, UK.

[2]Stephenson Institute for Renewable Energy, University of Liverpool, Peach Street, L69 7ZF Liverpool, UK.

[3]Leverhulme Research Centre for Functional Materials Design, Materials Innovation Factory, University of Liverpool, UK

*Corresponding Author: M.J.Rosseinsky@liverpool.ac.uk

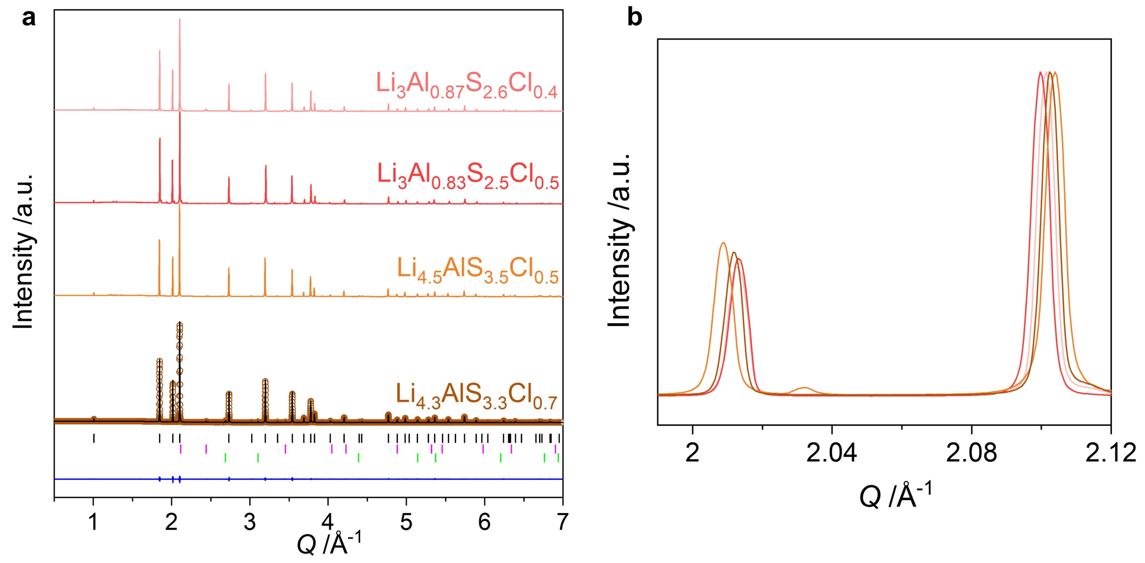

Figure S1. (a) SXRD patterns of the four "phase A" compositions along with a Le Bail fit against SXRD data of $Li_{4.3}AlS_{3.3}Cl_{0.7}$ ($P3m1$, a = 3.93161(3) Å and c = 6.23971(3) Å), with I_{obs} (dark orange dots), I_{calc} (black line), I_{obs}-I_{calc} (blue line), and Bragg reflections (black tick marks for $Li_{4.3}AlS_{3.3}Cl_{0.7}$, pink tick marks for LiCl (~ 2wt %) and green tick marks for Al (~ 1 wt %). (b) Zoom of the SXRD patterns of the four "phase A" samples showing the shift of the reflections with Q depending on the composition.

Table S1. Lattice parameters and cell volume of the four compositions showing phase A in high purity (space group $P\bar{3}m1$).

Composition	*a* / Å	*c* / Å	*V* / Å³
$Li_3Al_{0.87}S_{2.6}Cl_{0.4}$	3.92256(1)	6.2522(1)	83.311(2)
$Li_3Al_{0.83}S_{2.5}Cl_{0.5}$	3.92595(8)	6.2401(1)	83.404(5)
$Li_{4.5}AlS_{3.5}Cl_{0.5}$	3.93522(1)	6.2384(2)	83.664(3)
$Li_{4.3}AlS_{3.3}Cl_{0.7}$	3.93161(3)	6.2397(3)	83.528(2)

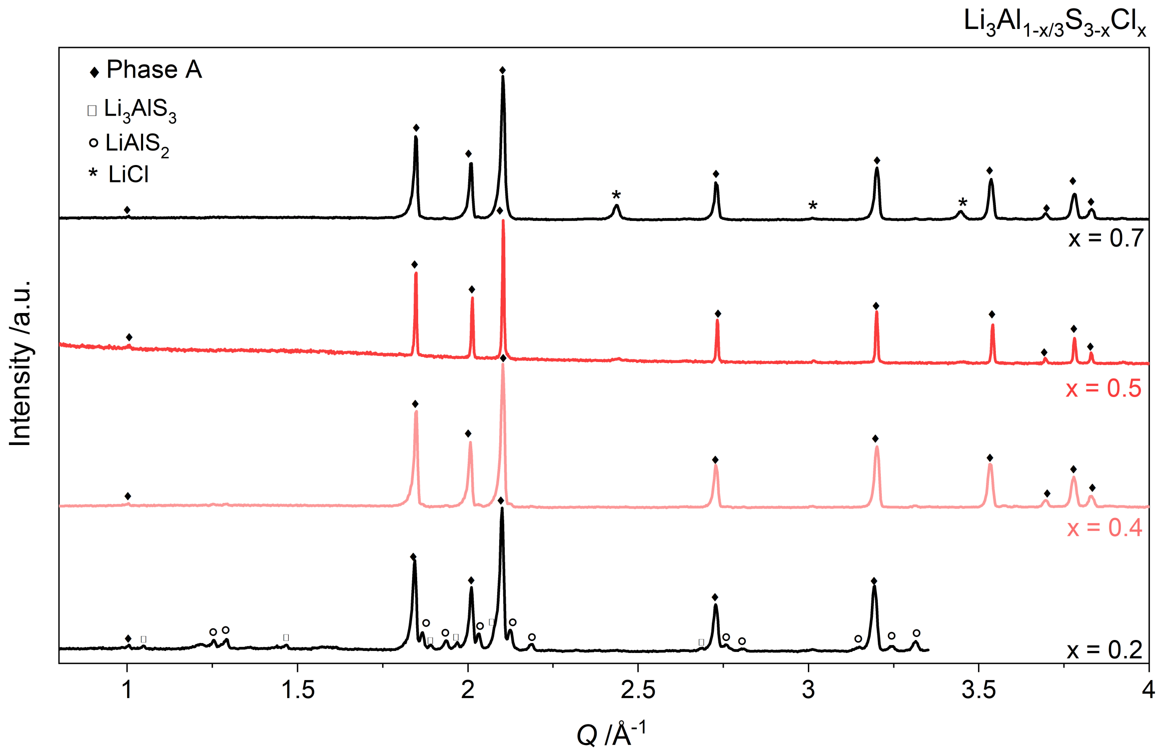

Figure S2. Laboratory XRD patterns of samples along the solid solution line: $Li_3Al_{1-x/3}S_{3-x}Cl_x$ (value of x in caption), λ = 1.5406 Å for x = 0.5 and λ = 0.70903 Å for the other compositions.

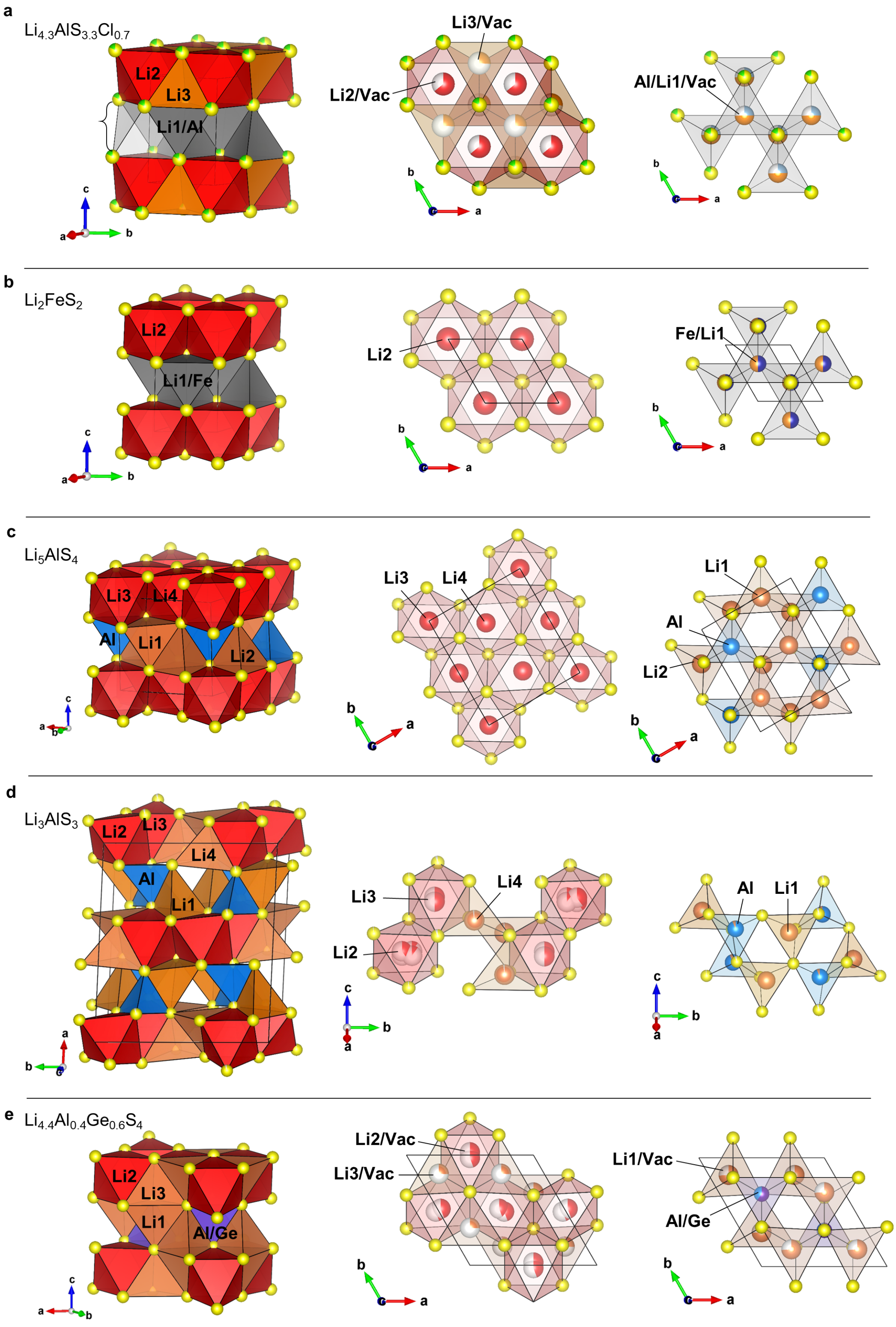

Figure S3. Comparison of the crystal structures of $Li_{4.3}AlS_{3.3}Cl_{0.7}$, Li_2FeS_2, Li_5AlS_4, Li_3AlS_3 and $Li_{4.4}Al_{0.4}Ge_{0.6}S_{4.4}$, showing a *hcp*-type packing of the anion sublattice, and different arrangement of the cations within the interstitial sites, forming two alternating layers: one "Li-only" polyhedral layer (middle figures) and one Li/*M* (*M* = Al, Fe, Ge) tetrahedral layer (right hand side figures). Color code for atoms and octahedra: Li octahedra (red); Li tetrahedra (orange); Al tetrahedra (blue); mixed Li/*M* tetrahedra (grey), mixed Al/Ge tetrahedra (purple), Fe atoms (deep blue), Ge atoms (mauve).

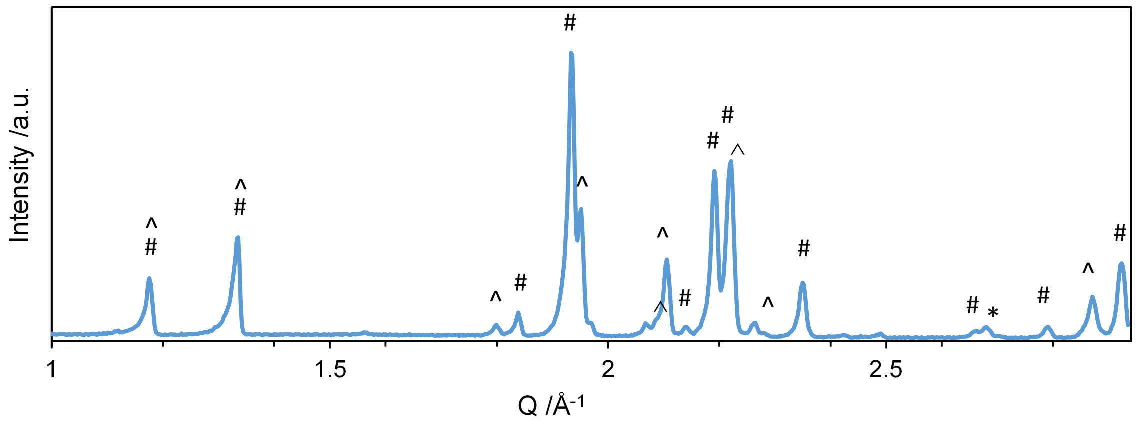

Figure S4. Laboratory XRD pattern of the Al_2S_3 starting material (Sigma Aldrich, 98 %), λ = 0.70932 Å showing the diffraction peaks corresponding to the α-Al_2S_3 hexagonal phase (^), the γ-Al_2S_3 trigonal phase (#) as well as the Al metal phase (*).

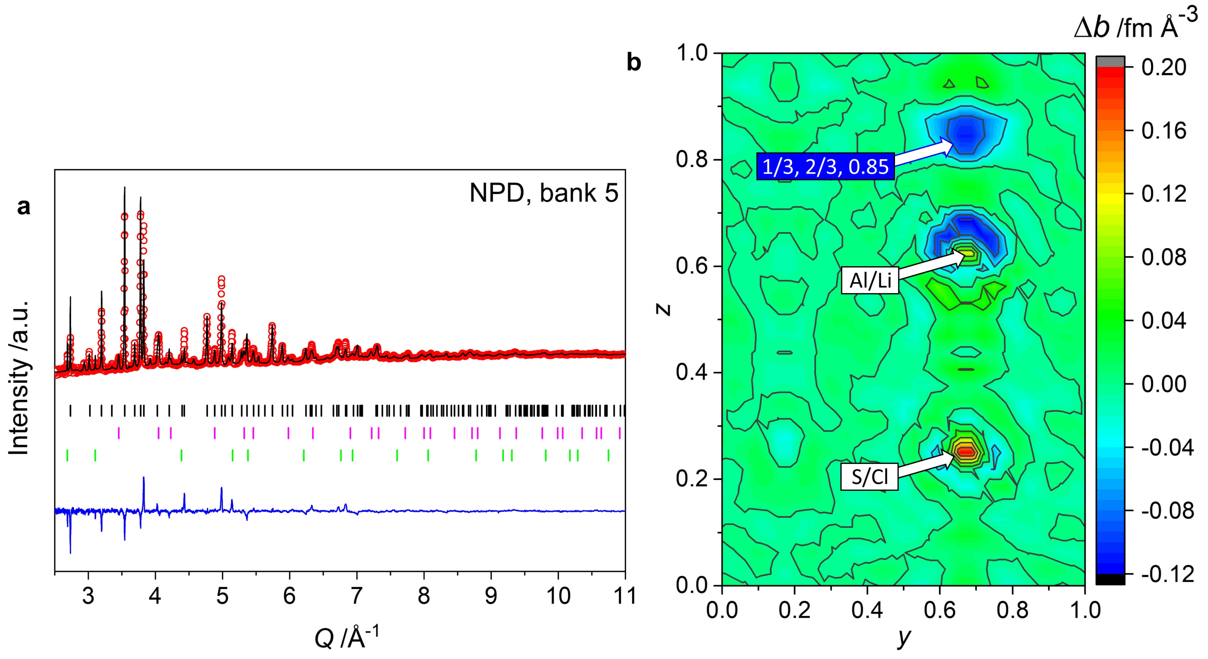

Figure S5. (a) Rietveld refinement of $Li_{4.3}AlS_{3.3}Cl_{0.7}$ against NPD data from Bank 5 of the Polaris instrument (ISIS, UK), using the Li_2FeS_2 structural model, showing the misfit, with χ^2 = 4.71. (b) Fourier difference map showing a residual negative scattering density at position (1/3, 2/3, 0.85), which could correspond to an extra Li position (^{7}Li has a negative scattering length: -2.22 fm).

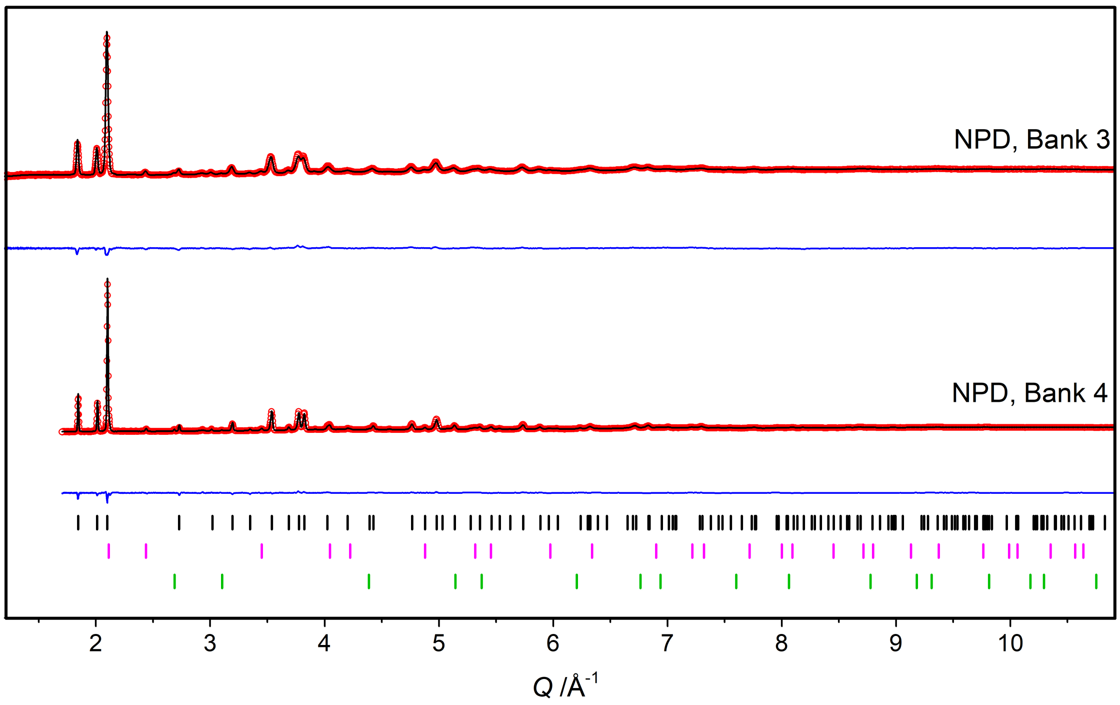

Figure S6. Final Rietveld fit against the NPD data from (a) Bank 3 ($2\theta = 52.210$ °), and (b) Bank 4 ($2\theta = 92.590$ °) of Polaris instrument (ISIS, UK), with I_{obs} (red dots), I_{calc} (black line), I_{obs}-I_{calc} (blue line), and Bragg reflections (black tick marks for $Li_{4.3}AlS_{3.3}Cl_{0.7}$, pink tick marks for LiCl (~2 w%) and green tick marks for Al (~1 w%).

Table S2. Summary of the outcome of the refinement against synchrotron X-Ray powder diffraction (SXRD) and neutron powder diffraction (NPD) data of $Li_{4.3}AlS_{3.3}Cl_{0.7}$.

Radiation	NPD bank 2	NPD bank 3	NPD bank 4	NPD bank 5	SXRD
Refined composition	$Li_{4.32(1)}AlS_{3.308(4)}Cl_{0.71(2)}$				
Formula weight ($g\cdot mol^{-1}$)	188.21				
Space group	$P\bar{3}m1$				
Z	2				
Density ($g\cdot cm^{-3}$)	1.863				
Temperature (K)	298				
Angle (°) / Wavelength (Å)	25.990	52.210	92.590	146.720	0.825186
d spacing range (Å)	0.5530 - 13.9632	0.5140 - 5.9272	0.4372 - 3.6916	2.6967 - 0.3575	0.5609 - 22.6882
TOF (µsec.) / 2θ (°) range	1206.8900 - 24886.6992	1106.3101 - 19938.3008	1106.2000 - 19933.8008	1103.4800 - 19938.5000	2.0840 - 92.1160
TOF (µsec.) / 2θ (°) step	7.8229	6.5118	6.5102	3.2536	0.004
No. of reflections	230	286	438	769	220

No. of data	3029	2894	2894	5791	22509
**No. of refine parameters**	16 (profile)<br>13 (atomic)				18 (profile)<br>13 (atomic)
a **(Å)**	3.93161(3)				
c **(Å)**	6.23971(3)				
**Volume (Å^3)**	83.528(2)				
R_p	15.1	12.9	14.8	21.8	7.20
R_{wp}	10.3	12.3	13.9	16.3	6.62
R_{exp}	3.34	4.36	5.27	12.27	1.85
R_{Bragg}	8.68	8.94	9.41	15.2	2.20
χ^2	9.43	8.01	6.93	1.77	12.9
$\rho_{min./max.}$ **residuals (fm/e$^-$·Å^{-3})**	-0.12 / 0.03	-0.04 / 0.03	-0.05 / 0.11	-0.05 / 0.18	-0.34 / 0.41

Table S3. Crystal structure of $Li_{4.3}AlS_{3.3}Cl_{0.7}$

Site	x	y	z	*B* (Å^3)	*sof*	Wyckoff position
S	1/3	2/3	0.2488(5)	2.54(3)	0.827(3)	2*d*
Cl	1/3	2/3	0.2488(5)	2.17(3)	0.178(7)	2*d*
Al	1/3	2/3	0.625(1)	2.0(2)	0.25	2*d*
Li1	1/3	2/3	0.668(3)	1.8(3)	0.499(2)	2*d*
Li3	1/3	2/3	0.872(7)	1.8(3)	0.260(2)	2*d*
Li2	0	0	0	4.2(4)	0.644(2)	1*a*

Table S4. Selected interatomic distances and angles for $Li_{3.3}AlS_{3.3}Cl_{0.7}$.

Atom	Distance around atom (Å)			Angles around atom (°)				Coordination
Al	Al	S	2.3454(2)	S	Al	S	109.176(12) ×3	4
	Al	S	2.4032(9) ×3	S	Al	S	109.764(13) ×3	
Li1	Li1	S	2.615(6)	S	Li1	S	102.899(12) ×3	4
	Li1	S	2.3286(13) ×3	S	Li1	S	115.166(13) ×3	
Li3	Li3	S	2.351(13)	S	Li3	S	108.367(12) ×3	4
	Li3	S	2.392(4) ×3	S	Li3	S	110.552(13) ×3	
Li2	Li2	S	2.7501(6) ×6	S	Li2	S	91.257(10) ×6	6
				S	Li2	S	88.744(9) ×6	
				S	Li2	S	180.000(15) ×3	

Table S5. Averaged calculated frequencies for the different *M-X* bonds (*M* = Li, Al; *X*= S, Cl) in Li_3AlS_3 and $Li_{4.3}AlS_{3.3}Cl_{0.7}$ considering the bond as a simple harmonic oscillator: $\nu_{th} = \frac{1}{2\pi} * \sqrt{k/\mu}$, with *k* the spring constant ($k = 2e^2/4\pi\varepsilon_0 d^3$), *d* the interatomic distance, e the electron charge, ε_0 the vacuum permittivity, and μ the reduced mass.

	**Li_3AlS_3**	**$Li_{4.3}AlS_{3.3}Cl_{0.7}$**
$\nu_{th}(Li\text{-}X)$ /cm^{-1}	300(1)	315(3)
$\nu_{th}(Al\text{-}X)$ /cm^{-1}	215(1)	197(1)

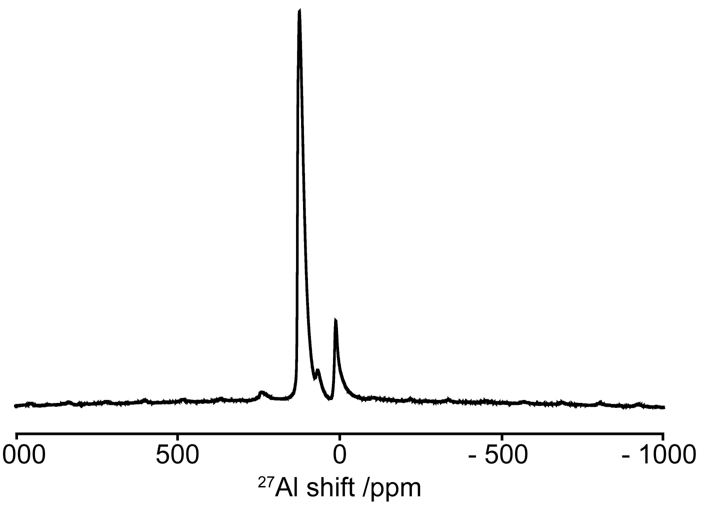

Figure S7. One-dimensional ^{27}Al MAS NMR spectrum of $Li_{4.3}AlS_{3.3}Cl_{0.7}$ obtained at a magnetic field of 20 T and under MAS at 20 kHz showing the full spectral width.

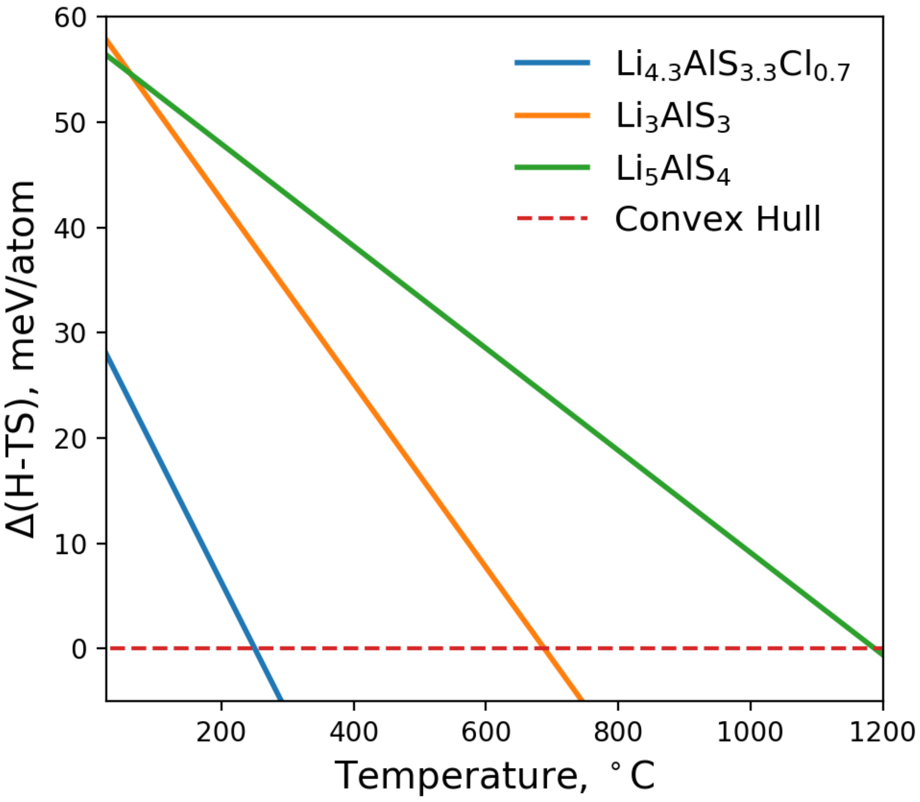

Figure S8. ΔG (= G_o- G_d) *vs.* temperature plots calculated for Li_5AlS_4, Li_3AlS_3 and $Li_{4.3}AlS_{3.3}Cl_{0.7}$ materials in their hypothetic ordered (G_o) and disordered (G_d) structure.

Table S6. Results of the fits to the impedance data collected at 30 °C on cylindrical pellets of $Li_{4.3}AlS_{3.3}Cl_{0.7}$ and $Li_{4.5}AlS_{3.5}Cl_{0.5}$. Admittance of the Constant Phase Element: $Y_{CPE} = Q * (\omega i)^n$, R stands for resistance, GB for Grain Boundary, and BE for Blocking Electrode. The value of the estimated capacitances were derived from the Constant Phase Element parameters using equation from Hsu and Mansfeld:[1] $C = Q * (\omega_{\text{max}})^{n-1}$, where ω_{max} is the frequency at the top of the considered semi-circle.

**Sample**	R_{bulk} **/Ω**	Q_{bulk}	n_{bulk}	$\omega_{max,bulk}$	C_{bulk}
$Li_{4.3}AlS_{3.3}Cl_{0.7}$	$4.8(3) \times 10^4$	$1.9(5) \times 10^{-12}$	1.0(1)	300 kHz	$8(4) \times 10^{-12}$ F
$Li_{4.5}AlS_{3.5}Cl_{0.5}$	$5.3(1) \times 10^4$	$3.5(6) \times 10^{-11}$	0.90(1)	300 kHz	$1.0(4) \times 10^{-11}$ F

**Sample**	R_{GB} **/Ω**	Q_{GB}	n_{GB}	$\omega_{max,GB}$	C_{GB}	Q_{BE}	n_{BE}
$Li_{4.3}AlS_{3.3}Cl_{0.7}$	$8.2(6) \times 10^4$	$3.4(6) \times 10^{-8}$	0.54(3)	6000 Hz	$6(3) \times 10^{-10}$ F	$8.7(3) \times 10^{-6}$	0.18(1)
$Li_{4.5}AlS_{3.5}Cl_{0.5}$	$9.1(2) \times 10^4$	$2.6(3) \times 10^{-7}$	0.52(2)	200 Hz	$2.1(3) \times 10^{-8}$ F	$8.2(1) \times 10^{-6}$	0.63(1)

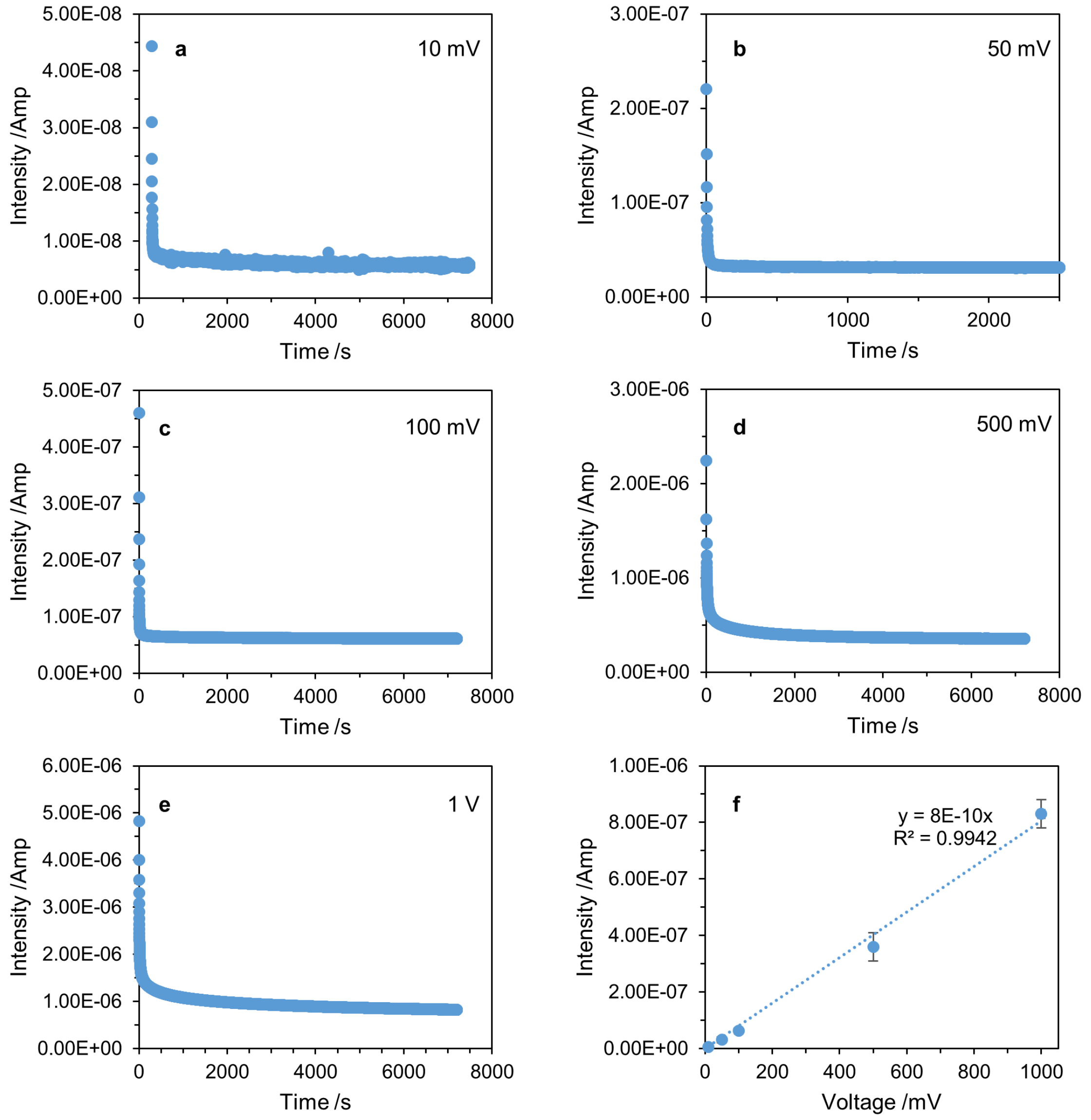

Figure S9. Dc-polarization measurement performed on a cylindrical sintered pellet of $Li_{4.5}AlS_{3.5}Cl_{0.5}$ of 0.9 mm thickness and 4.8 mm diameter. **a-e**: current versus time measurement for different applied voltage (in caption) showing that the current first accounts for ionic and electronic contribution, and decreases exponentially as the ions polarize at the electrode interface. The residual current, noted I_∞, is then only comprising the electron flow. **f**: residual current I_∞ *vs.* potential curve showing the ohmic behavior of the electron flow, with an electronic conductivity $\rho_e = I_\infty/U * e/S$, where e is the thickness and S the surface of the pellet. We obtain $\rho_e = 8(1) \times 10^{-10}$ S·cm^{-1}, which corresponds to 0.010(2) % of the total conductivity of the sample.

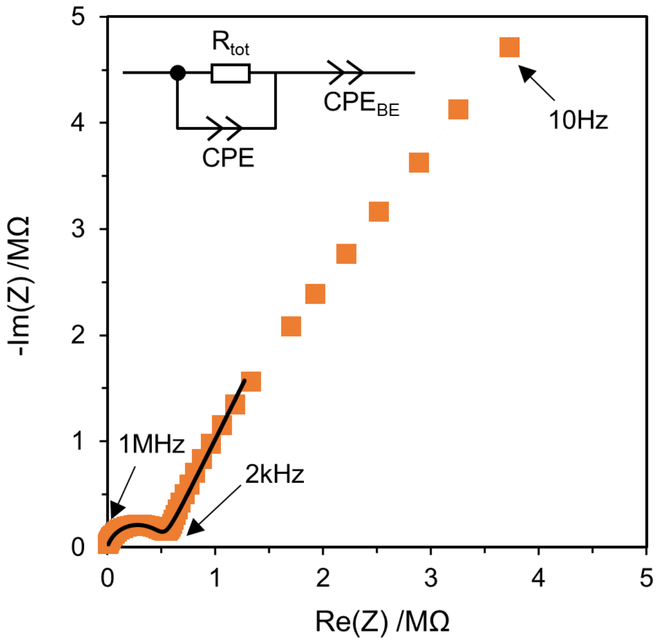

Figure S10. Room temperature (303 K) Nyquist plot of a $Li_{4.3}AlS_{3.3}Cl_{0.7}$ pellet before sintering and the fit using the equivalent circuit in inset (black line). The fit leads to a measured total conductivity of the pellet before sintering of 1.3(4) $\times 10^{-6}$ S·cm^{-1}. The increase of the total conductivity of the sample after the sintering treatment (equal to 2.5(2) $\times 10^{-6}$ S·cm^{-1}) can be attributed to the reduction of the grain boundary resistance.

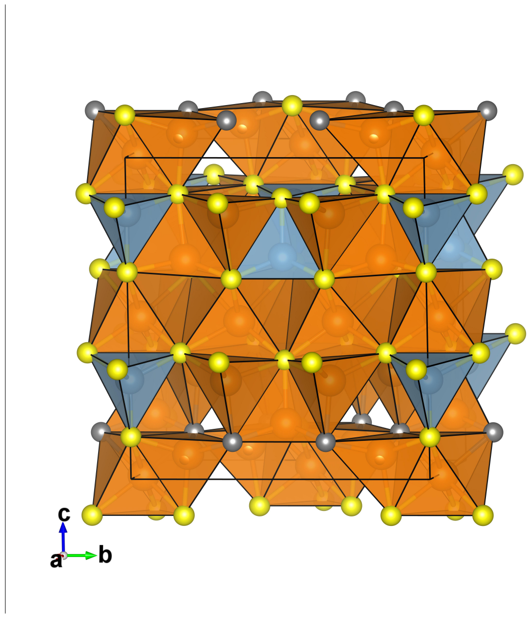

Figure S11. The relaxed model structure for $Li_{13}Al_3S_{10}Cl_2$ generated from the disordered experimental structure and used for AIMD calculations. Al tetrahedra are shown in blue, Li tetrahedra and octahedra in orange, sulfide ions in yellow and chloride ions in grey.

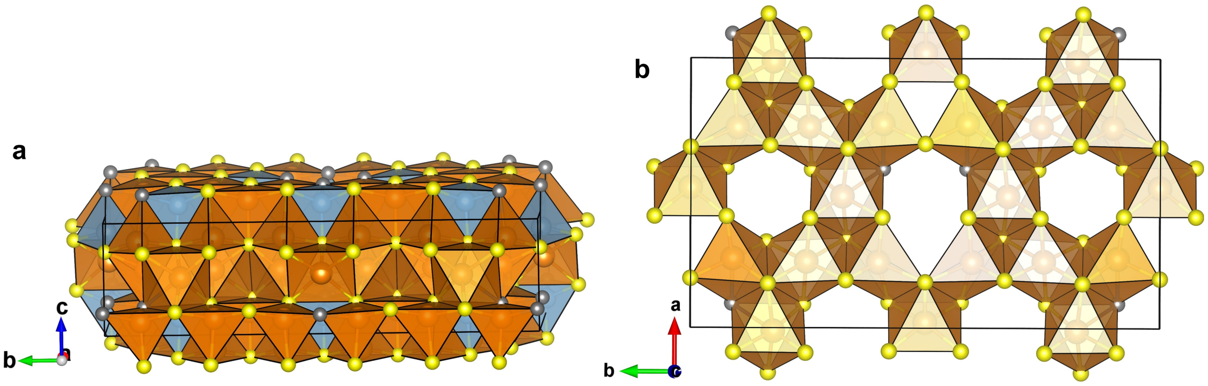

Figure S12. The relaxed model structure of $Li_{13}Al_3S_{10}Cl_2$ with cation ordering matching the experimental structure of $Li_{4.4}Al_{0.4}Ge_{0.6}S_4$ and used for AIMD calculations. Al tetrahedra are shown in blue, Li tetrahedra and octahedra in orange, sulfide ions in yellow and chloride ions in grey. The panel on the right shows the octahedral layer with the ordered vacant octahedral sites present in $Li_{4.4}Al_{0.4}Ge_{0.6}S_4$.[2]

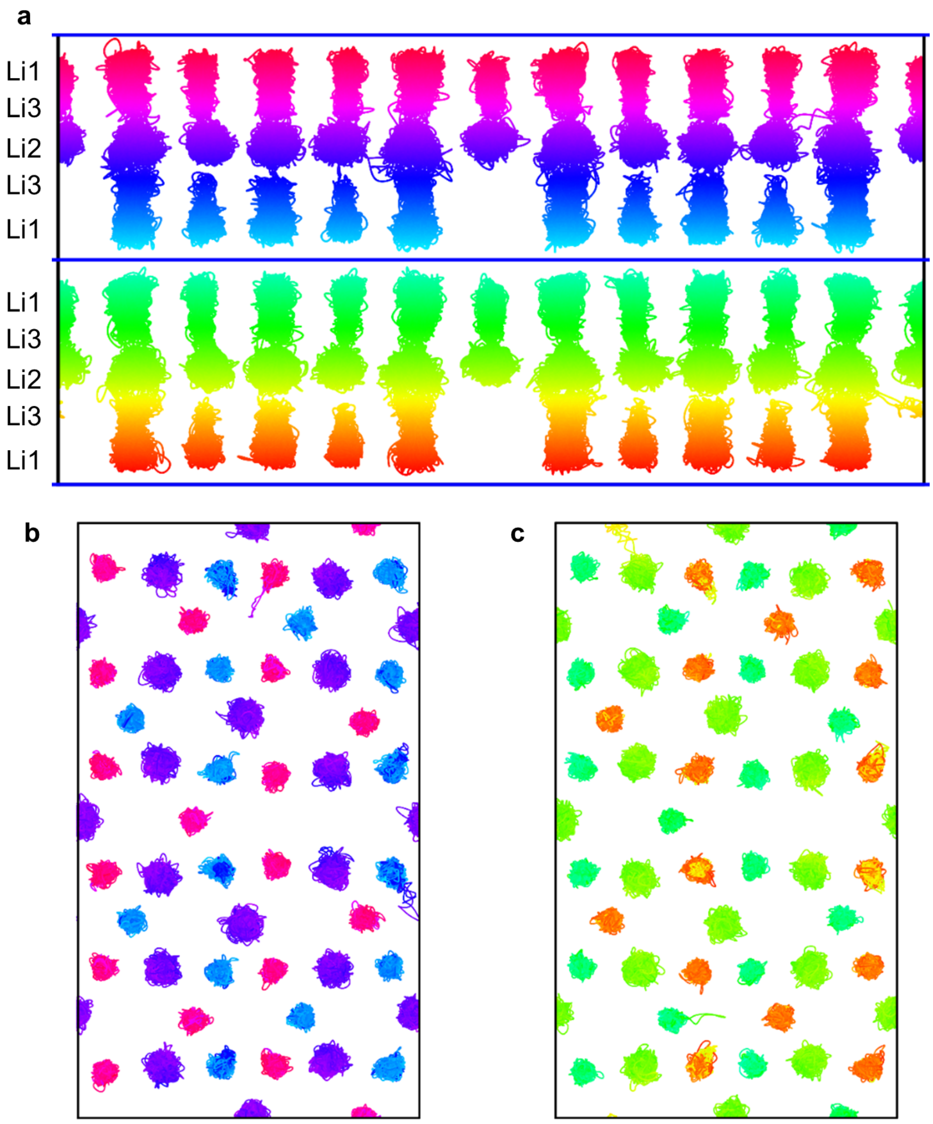

Figure S13. The position of Li ions within the $Li_{104}Al_{24}S_{80}Cl_{16}$ supercell of $Li_{13}Al_3S_{10}Cl_2$ with cation ordering matching the experimental structure of $Li_{4.4}Al_{0.4}Ge_{0.6}S_4$ over a 100 ps AIMD trajectory. Atoms are colored according to their position along the *c* axis. This can clearly be seen in (a), viewed along the *a* axis. In panels (b) and (c) the cell is viewed down the *c* axis. The cell is split into two halves. Panel (b) shows the top half of the cell and panel (c) shows the bottom half of the cell.

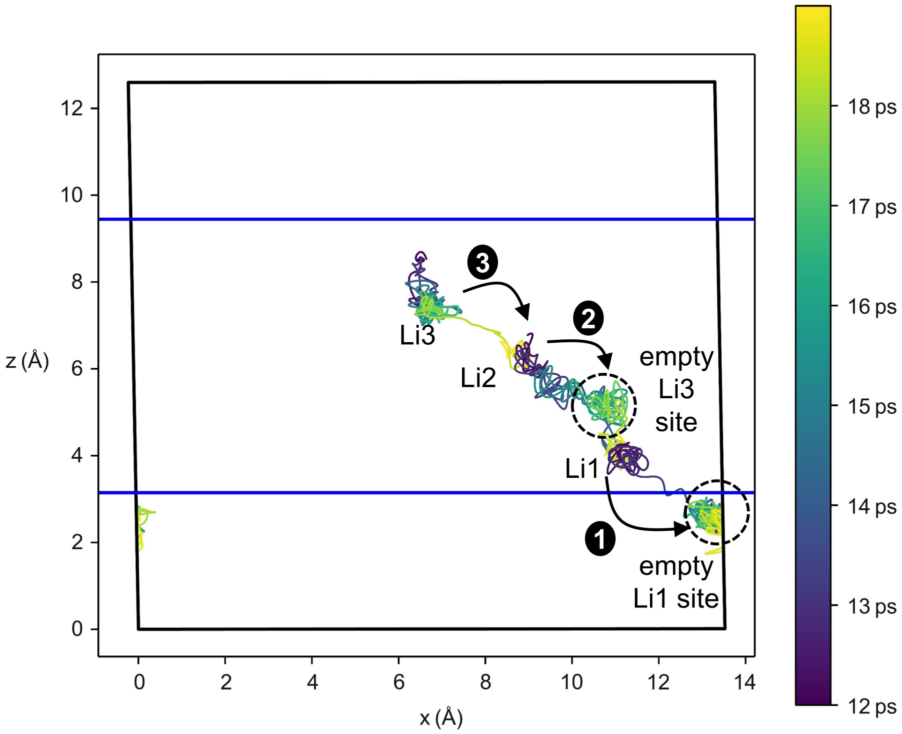

Figure S14. Paths of three adjacent Li ions (in Li1, Li2 and Li3 positions) in the time period 12-19 ps showing that Li1-Li1 hop happen first (~13 ps, purple), followed by Li2-Li3 (~15 ps, blue) and Li3-Li2 hops (~18 ps, green).

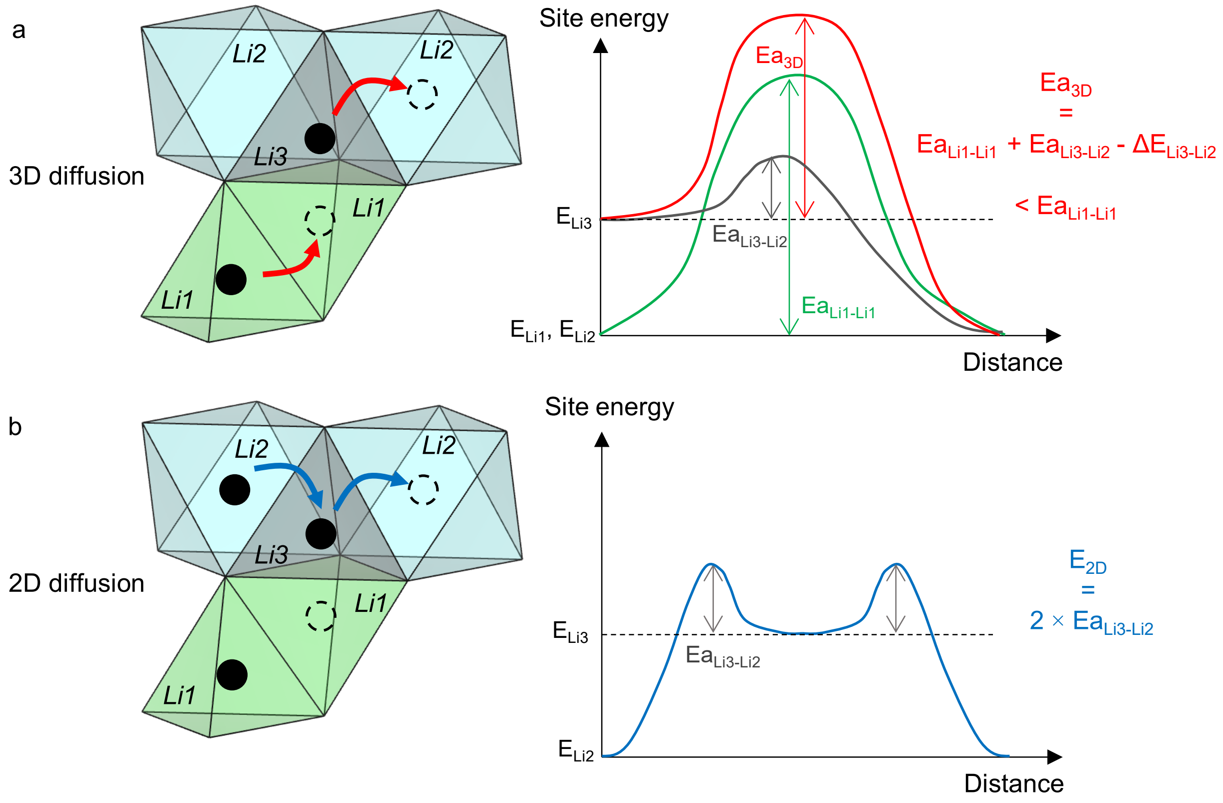

Figure S15. Activation energies involved in the 3D (a) and 2D (b) diffusion processes in $Li_{4.3}AlS_{3.3}Cl_{0.7}$ (only energies of the initial and final states are considered for simplification). 3D diffusion is triggered thanks to a concerted migration mechanism decreasing the apparent activation energy for Li1-Li1 hops.

References

(1) Hsu, C. H.; Mansfeld, F. Technical Note: Concerning the Conversion of the Constant Phase Element Parameter Y0 into a Capacitance. *Corrosion* **2001**, *57* (09).

(2) Leube, B. T.; Inglis, K. K.; Carrington, C.-G. D. of L.; Sharp, P. M.; Shin, J. F.; Neale, A. R.; Manning, T. D.; Pitcher, M. J.; Hardwick, L. J.; Dyer, M. S.; Blanc, F.; Claridge, J. B.; Rosseinsky, M. J. Lithium Transport in $Li_{4.4}M_{0.4}M'_{0.6}S_4$ ($M = Al^{3+}$, Ga^{3+}, and $M' = Ge^{4+}$, Sn^{4+}): Combined Crystallographic, Conductivity, Solid State NMR, and Computational Studies. *Chem. Mater.* **2018**, *30* (20), 7183–7200.
